# Willingness to pay for health insurance in the informal sector of Sierra Leone

**DOI:** 10.1371/journal.pone.0189915

**Published:** 2018-05-16

**Authors:** Mireia Jofre-Bonet, Joseph Kamara

**Affiliations:** 1 Department of Economics; City, University of London, London, United Kingdom; 2 LSE Health Policy, London School of Economics and Political Science, London, United Kingdom; 3 National Social Security and Insurance Trust, Ministry of Labour and Social Security, Freetown, Sierra Leone; Johns Hopkins University Bloomberg School of Public Health, UNITED STATES

## Abstract

**Purpose:**

The objective of this project is to study the willingness to pay (WTP) for health insurance (HI) of individuals working in the informal sector in Sierra Leone, using a purposely-designed survey of a representative sample of this sector.

**Methods:**

We elicit the WTP using the Double-Bounded Dichotomous Choice with Follow Up method. We also examine the factors that are positively and negatively associated with the likelihood of the respondents to answer affirmatively to joining a HI scheme and to paying three different possible premiums, to join the HI scheme. We additionally analyze the individual and household characteristics associated with the maximum amount the household is willing to pay to join the HI scheme.

**Results:**

The results indicate that the average WTP for the HI is 20,237.16 SLL (3.6 USD) per adult but it ranges from about 14,000 SLL (2.5 USD) to about 35,000 SLL (6.2 USD) depending on region, occupation, household and respondent characteristics. The analysis of the maximum WTP indicates that living outside the Western region and working in farming instead of petty trade are associated with a decrease in the maximum premium respondents are WTP for the HI scheme. Instead, the maximum WTP is positively associated to being a driver or a biker; having secondary or tertiary education (as opposed to not having any); the number of pregnant women in the household; having a TV; and, having paid for the last medical requirement.

**Conclusions:**

In summary, the various analyses show that a premium for the HI package could be set at approximately 20,000 SLL (3.54 USD) but also that establishing a single premium for all individuals in the informal sector could be risky. The efficient functioning of a HI scheme relies on covering as much of the population as possible, in order to spread risks and make the scheme viable. The impact of the various population characteristics raises the issue of how to rate premiums. In other words, setting a premium that may be too high for a big proportion of the population could mean losing many potential enrollees and might have viability consequences for the operation of the scheme.

## Introduction

Sierra Leone has a population of 6.6 M, with an average life expectancy of 56.1 years, despite 41% of the population being under 15 and only 3.7% above 65. Around 90% of the labor force in Sierra Leone works in the informal sector, mostly subsistence or other small-scale agriculture. The gross domestic product (GDP) per capita in Sierra Leone is about 490.56 [1]. This extremely low GDP per capita fuels the permanent fiscal deficit of the government, which is aggravated by the existence of pervasive corruption. The result is that access to health care and education in Sierra Leone is particularly poor. Health care treatments often rely on NGOs, the goodwill of doctors and nurses, and require unaffordable out-of-pocket payments from the population. This situation has become especially problematic in light of the Ebola crisis of 2014–2015, which killed around 4,000 persons and may have caused the GDP of Sierra Leone to drop by 21% [2]. In addition, currently Sierra Leone has an extremely high child mortality rate and one of the highest maternal mortality rates in the world in part explained by poor access to health care services at the time of giving birth [3]. In view of this, there is an interest from the government to explore alternatives that would ensure access to health care for the population of Sierra Leone through a social insurance scheme. The purpose of this study is to investigate the willingness to pay (WTP) of the informal sector for a health insurance (HI) scheme on a basic health care package, including primary and selected secondary health care services. This study aims to study the feasibility of introducing a social insurance scheme to ensure better access to health care. This aims to guarantee better access to health care and avoid health crises such as that of Ebola, which ended up being not only causing thousands of avoidable deaths and denting Sierra Leoneans’ health and livelihood, but also becoming a threat to global health.

The remainder of this paper is organized as follows: the subsequent section provides a brief summary of the background and outlines the methods used; section 3 presents the survey used to elicit the WTP; section 4 discusses the results of the empirical analysis, including the factors that are associated with the likelihood of accepting to pay for the HI scheme; section 5 discusses the findings and their reliability, as well as potential lines of future work; and section 6 reflects on the implications of the findings.

## Background and methods

There are different ways to estimate the WTP for goods and services. One approach is the revealed preferences, which is based on observing actual individuals’ purchasing behavior at different prices. The second method is based on stated, rather than revealed, preferences to elicit the WTP for goods and services that are often not yet marketed. Given the inexistence of HI schemes in most low and medium income countries, the stated preferences’ approach has been used extensively in the HI framework. Particularly, the most well-known stated preferences method is contingent valuation (CV), which involves asking individuals how much they are willing to pay for the provision of a given good or service, given a detailed description of what is on offer. CV has been validated as a method to obtain reliable estimates for not yet marketed goods and services [4,5,6].

### Background

There exists an extensive literature on WTP for HI, applying CV methods [4]. Nosratnejad et al. (2016) provide a thorough and up-to-date Systematic Review of the Willingness to Pay for Health Insurance in Low and Middle Income Countries, to which we refer to here [5]. This review reports country, year, population, if it is at the household or at the individual level and sample size, and also the measurement method of the WTP for each included study. The work of Nosratnejad et al. (2016) also calculates the WTP for HI as the percentage of the GDP per capita and of the net national income per capita [5]. Given the up-to-date and comprehensive nature of this review, we do not attempt its replication and use this information as the basis of comparison with our own analysis.

The CV method can follow three different general approaches to elicit answers from individuals: using their bids; presenting them with take-it-or-leave-it questions; or applying dichotomous choice methods. The first general approach is associated with open-ended questions, in which the individual is asked how much is he/she is willing to pay for a good or service that has been previously described along with a hypothetical scenario. The second approach usually involves payment cards; the individuals are presented with a series of amounts for possible payments and they chose the one that is closer to their individual valuation. The last approach is to use dichotomous choice questions, which, in their simplest form, ask the individual if he/she would be willing to pay an amount X for a described hypothetical good or service. For instance, the studies included in Nosratnejad *et al*.‘s systematic literature review elicit WTP for HI by means of either bidding games, open-ended questions, payment cards, take-it-or-leave-it questions, or double-bounded dichotomous choice [5].

Nosratnejad et al. (2016) [5] find that the average WTP of individuals amounted to 1.18% of the GDP per capita or 1.39% of adjusted net national income per capita across the range of identified studies. They also conclude that factors such as family size, education level, and income are correlated with higher WTP for HI, while age correlates negatively with WTP for HI. Given the small number of high values, for example associated with China, the median is arguably the better description of the findings, and the median WTP of individuals was 1.9% of the GDP per capita or 2.3% of adjusted net national income per capita across the range of identified studies.

### Methods

In this study, we present the results of eliciting the WTP for HI in the informal sector in Sierra Leone applying the double-bounded dichotomous choice with follow up method. As described in detail by López-Feldman [6] and Hanemann et al. [7], the simplest form of the double-bounded dichotomous choice method entails asking an individual if he/she would pay a given amount, F_i_, to obtain the goods and services. This, under some assumptions (i.e., linearity), allows to express the WTP as a function of the individual and/or household characteristics. The individual will answer yes to the question only if his/her WTP is greater than the suggested fee—or premium in the case of HI—F_i_. Therefore, assuming normality, the probability of having a positive observation to the question can be expressed as a function of the individual and/or household characteristics. This allows for two types of analyses. First, identifying the characteristics influencing the probability of accepting the HI scheme at different premium levels. Second, the overall average WTP and the WTP for specific population subsamples.

In this work, we use a refinement of this method, referred to as the double-bounded choice with follow-up. Following this approach, the individual is asked if he/she is willing to pay an amount F_i_ to get access to, in our case, an HI scheme. If the individual answers “no”, the same question is asked again, but the given amount is set at a lower level. If the individual answers “yes”, then the next question sets a higher amount [6,7]. Note that, in this method, the second question depends on the answer obtained for the first question. As such, this method provides us with two answers for each individual based on the initial suggested amount, followed by the revised (downwards or upwards) follow-up question. Estimation of the WTP is slightly more complicated due to the dependency of the second amount offered on the initial offer. In the simpler case, with the double-bounded choice with follow-up, we are able to calculate which personal characteristics make it more likely to take up the HI scheme and, also, the WTP for insurance itself. As illustrated for instance in the systematic review of Nosratnejad et al. [5], the double-bounded choice with follow-up method has been validated and widely used to elicit WTP for health insurance schemes in developing countries. In the subsequent section, we explain how the survey used to elicit the WTP for HI in Sierra Leone was designed and the double-bounded dichotomous choice with bounded follow-up applied to this particular case.

Besides eliciting the WTP applying the double-bounded dichotomous choice with follow-up method, we run several types of complementary analysis: First, in order to identify which factors significantly increase or decrease the likelihood of a respondent to say YES to the question on whether he/she would join or not the health insurance scheme in general, we apply a Probit Model, which is used to estimate a model involving a binary dependent variable [8]. We also use Probit Models to estimate which factors are associated to answering yes to joining the HI scheme at different premium levels. Second, to examine what factors influence, and in which direction, the maximum amount individuals declare they would be WTP to join a health insurance scheme we apply a TOBIT model, which estimates linear relationships between variables when there is either left or right censoring. In this case, the censoring of the maximum willingness to pay is at 0 [8].

## Willingness to pay survey

The data we use to elicit the WTP for HI regarding the population in the informal sector in Sierra Leone was obtained using a purposely-designed survey described in the supplementary information section. The final ethical approval of the survey was granted by the Technical Committee for the Sierra Leone Social Health Insurance (SLeSHI) Scheme, which is a board in charge of approving the Ethics of any project supported by the institution. It was required by the Technical Committee that the field workers would be trained to seek each respondent's consent before administering the survey. The informed consent was written but read verbally to the respondents because the majority of them could not read or write. The explanation of the nature of the survey and the right to choose to participate or not was done in the respondent’s own local dialect. If the respondent chose not to participate, this was recorded and he or she was thanked.

As sample sizes as low as 250–400 have been shown to be sufficient for the double and single double bound contingent valuation methods to perform well in well in giving point estimates for the parameters and of the mean WTP [9,10], we ensured we had a sample size of at least 1,400 households in each of the four regions. To select the survey sample, first, a three-stage stratified sampling method was used to identify survey respondents. To do so, the population census enumeration area and the provincial census of Sierra Leone for the year 2015 were used. The first stage involved assigning a sample size to each of the 14 districts in the country. Using the population of each district, a sample size for that district was estimated, and, from this, the total sample size per district was obtained. The second stage involved assigning a sample size to each of a number of chiefdoms. There are 149 chiefdoms in the country. In the third and final stage, villages and respondents within a village were randomly selected to fulfill the sample size requirements for each chiefdom and across chiefdoms for each district. The survey was subsequently administered in each chiefdom headquarter town by a team of 54 enumerators who were supervised by 12 leaders. As a result of this process, a total of 10,080 questionnaires were administered to all 14 districts within the four main regions of Sierra Leone and the data double entered.

Besides the contingent valuation questions and the maximum WTP for the health insurance scheme, the survey contained questions on household and respondent socio-economic characteristics (including income and occupation), health of the household, use of health care providers in the last three months, questions on perceived health care quality of the health care facilities used, distance to the nearest health care center, difficulty in paying for health care use, and other essential information. We summarize the answers in the following section and present the survey in full in S1 and S2 Inserts in the supplementary information.

Most significantly, the survey asks the respondent to reveal if he/she would be willing to join the described HI scheme (described in document S1 Insert), as well as the reasons why they answer yes or no to that question. The respondent also answers the following questions with regard to the elicitation of their WTP for such an HI scheme. Table 1 below reports the specific WTP questions contained in the survey.

**Table 1:** WTP for HI questions.

Assuming you were to pay 20,000 SLL per month per head as premium for the SLeSHI Scheme, will you be willing to pay? Yes ☐ No ☐ Don’t know ☐
If the premium is set at 30,000 SLL per month per head for the SLeSHI Scheme, would you be willing to pay? Yes ☐ No ☐ Don’t know ☐
If the premium is set at 10,000 SLL per month per head for the SLeSHI Scheme, would you be willing to pay? Yes ☐ No ☐ Don’t know ☐

The chosen bidding structure relies on a previous study [10], which had elicited the WTP of the informal sector in Sierra Leone using a Discrete Choice Experiment. This work provides arguments for the value of 20,000 SLL (USD 3.54) as the initial premium for the health insurance scheme. The other two values, 30,000 (USD 5.31) SLL and 10,000 SLL (USD 1.77), which subtract and add 10,000 SLL to the initial bid, are also in concordance to the range of values obtained in the previous work.

## Empirical analysis

### Descriptive statistics

S1 Table contains the detailed summary statistics for the full sample. As indicated by this table, all four regions of the country are well represented, although the Western region dominates responses with 38% of all observations, the Eastern region represents 18%, the Northern 27%, and the Southern 17%. The respective populations for the regions are Western, 1,493,243; Eastern, 1,641,012; Northern, 2,502,805; and Southern, 1,438,572. As such, the Western region is slightly over-represented, while the Eastern and Southern regions are slightly under-represented. Given the large size of the sample, the three-stage stratified sampling method applied and the methods of analysis we use, the slight difference in the relative size of each region’s sample is not a concern for the power of our findings.

**Fig 1:**
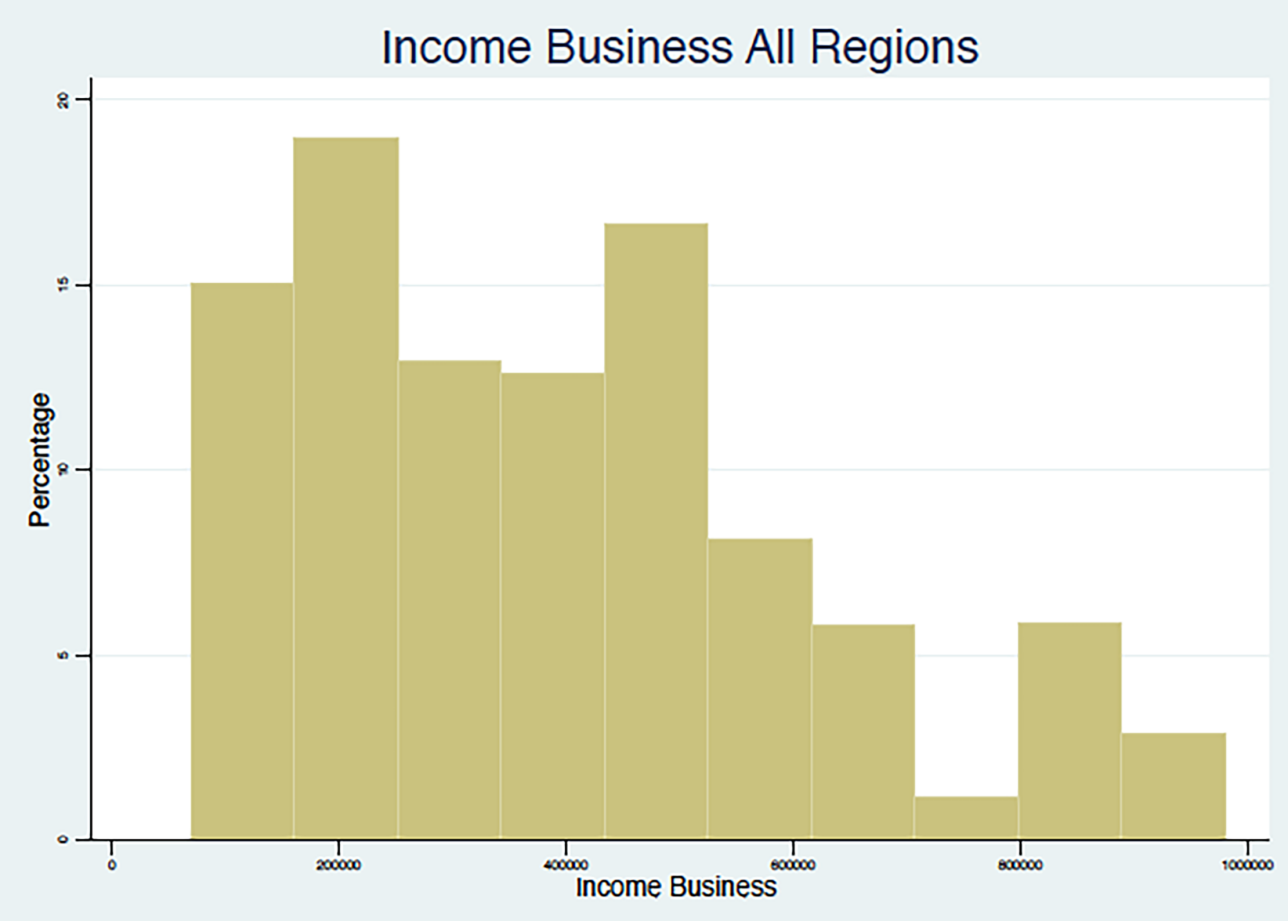
Income business for all regions.

The age group most represented in the sample is that ranging from 31 to 45. While it is not the largest section of the population, this is a major part of the current productive population, both in terms of employment and child-rearing. As such, this is a representative sample. Males account for 72%. The respondents are mostly in a monogamous marriage (58%). The most prevalent level of education is none (34%), although there are 20% of respondents with primary education, 19% with junior education, 16% with secondary, and 5% with tertiary education. The most common occupation is petty trade business (40%), followed by farming (20%). Although the mean monthly income from business is around 408,935 SLL (or about 74 USD), with a similar median (400,000 SLL), Fig 1 below shows that its distribution is skewed towards the lower income levels, which will be an important factor to take into account in assessing WTP for HI.

In terms of household characteristics, the household size is, on average, six members, the average number of rooms in the house is three, but can range up to 48. Seventy-three percent of the households have a kitchen, 96% a radio, and 87% a TV. The most common roof is corrugated and the type of house-wall material is mud or cement.

#### Descriptive statistics for the health insurance scheme questions

Participants are extremely willing to join such a scheme in all regions, but especially in the Northern one where 93% responded positively as per Table 2.

**Table 2:** Would you be willing to join the HI scheme?

Region	No	Yes	Total
Eastern	222	1,472	1,694
	13%	87%	
Northern	174	2,428	2,602
	7%	93%	
Southern	219	1,425	1,644
	13%	87%	
Western	462	3,084	3,546
	13%	87%	
Total	1,077	8,409	9,486
	11.35%	88.65%	

Note: The top number in each cell represents the number of respondents, and the bottom one the percentage of respondents in that region.

Table 3 reports the percentage of respondents that answer YES and NO to the second (30,000SLL) and third (10,000 SLL) premium choices for the health insurance scheme, conditional on their answer to the first question on whether they would join the scheme at a premium of 20,000 SLL.

**Table 3:** Percentage of responses.

	Second Premium			
First Premium	30,000 SLL (4,610 obs.)		10,000 SLL (7,251 obs.)	
20,000 SLL ()	Yes	No	Yes	No
Yes	41%	47%	45%	4%
No	3%	9%	32%	20%

Note that 4% of respondents declare they are willing to pay 20,000 SLL for the HI scheme and then decline the same scheme for 10,000 SLL. Equally, there are 3% of respondents willing to pay 30,000 SLL and not willing to pay 20,000 SLL. These may, of course, still be rational responses if the individuals are expressing their WTP as a valuation of the health package benefits. Nonetheless, such responses should be treated with caution and it is supportive for the general method that these responses represent such a low percentage of the total. Further, the respondents are also asked to answer, “What is the Maximum Amount will you be willing to pay per head based on your choice mode of payment?” Table 4 below, provides a summary for the answers.

**Table 4:** Average maximum WTP for the HI scheme.

Maximum WTP (8,136 observations)		
	Percentage	Mean (SLL)	Median (SLL)
WTP = 0	1.4%	0	0
WTP> 0	91.6%	20,802	15,000
Total	100%	20,766	15,000

Notes: W = 0 means that the Willingness to Pay for Health Insurance is zero. WTP>0 that it is positive. There are two observations with the maximum amount over 500,000 SLL we exclude from our calculations, as the WTP is above the declared income. The exchange rate (0.000177 SLL/USD) was obtained from http://www.xe.com/currencyconverter/convert as of 11/2016.

### Results

In the following subsections, we present four different sets of results. First, we describe the responses to the health, health care, and HI questions. Subsequently, we present the analysis of what characteristics make it more likely to answer yes to the question on the willingness to join the HI scheme. Then, we analyse which factors make it more likely to answer yes to the questions of whether the individual would join if the cost of the HI scheme was 20,000 SLL, 30,000 SLL or 10,000 SLL. Next, we report the WTP for the HI scheme, aggregately, and also by types of individuals. Finally, we examine which characteristics are associated with an increased or decreased maximum WTP for the HI scheme.

#### Univariate and bivariate descriptive analyses of health, health care, and health insurance questions

The analysis of the Health and Health Care related questions in S2 Table, reveals that only 5% of the respondents declare living in a household in very poor health, while 20% declare their household is in poor health, 43% medium health, 29% good health, and as little as 3% in a household in very good health. Therefore, even if around 68% of the respondents are in households with medium health or worse, only 22% declare that there is someone with a chronic illness or disability. Sixty-eight percent of the respondents report that, in the past three months, their household had at least one episode of illness. The most common choice for treatment was a public health care center (31%) or a public hospital (30%). The rest declare they got treatment from the local drug vendor (17%), a private facility (6%), or a traditional healer (3%). Fifteen percent provided self-treatment. The reasons for the treatment choice are access (49%), being cheap (27%), effectiveness (17%), followed by not being crowded (3%), personnel being courteous (3%), or other.

Forty-one percent of the respondents declare not having had treatment. The reasons why they chose this option are as follows: 41% respond that it was a self-limiting illness, 32% that they did not have the money to pay for the treatment, 13% affirm the health care facility is too far, and the rest mention not enough time or other causes. With respect to satisfaction from the cost and treatment, 48% are satisfied, 6% very satisfied, 18% are neutral, 21% are dissatisfied, while only 7% are very dissatisfied. When asked about the quality of the health care service in the area, only 27% respond with high quality or very high quality, 32% assess it to be neutral, 31% declare that it is low, and 10% very low.

Over half (53%) of respondents declare having had difficulties to pay the (medical) treatment and 38% found it very difficult to pay. The average of what they had to pay is 165,576.7 SLL (or about 29.80 USD), or just below half of their monthly income. We check how the average of the medical costs varies with their answer to the question on how they managed to pay: those that paid themselves (82%) declared having paid 166,042 SLL on average; those that had the family paying report average costs of 185,704.3 SLL; those that had the government paying, 114.513 SLL; if the community paid, the average is 156,904 SLL; and, finally, those that paid using other sources, paid an average of 77,741 SLL. As per the paying source for those that had difficulties, 40% had to use their savings, 19% borrowed, 16% accepted financial help from relatives, 16% had to work extra, 2% sold assets, and 6% cut down other expenses. Overall, 31% of respondents declared having to borrow from their relatives.

**Health Services available:** As summarized in S3 Table, forty-two percent of the respondents affirm the closest conventional health center is a health center, 14% a private clinic, and 45% a hospital. The average number of minutes they have to invest to reach the health center is 25.18, but the standard deviation is extremely large. With respect to how they access the health care centers, motorbike is the most common means of transport (53%), followed by public transport (17%) and other transport (25%). Vehicles (3%) and ambulance (0.5%) are seldom used.

**Health Insurance Scheme:** As reported in Table 5 below, 89% of the sample responds they would join the HI scheme they are presented with. The reasons they give for joining are (easier) access to medical care (50%), help others (11%), increased safety (33%), and ill health (5%).

**Table 5:** Health insurance variables.

		Obs	%
Would you be willing to join the HI described?	Yes	8,409	89%
	No	1,077	11%
If yes, why would you join SLL HI?	Access medical care	4,422	50%
	Help others	960	11%
	Safety	2,951	33%
	Ill health	472	5%
	Other reasons	17	0%
If not, why would not join the SLL HI?	Not enough money	641	32%
	No need	311	15%
	Other options are better	561	28%
	No trust in government	374	19%
	No HF in this area	27	1%
	No qualified heath facility	11	1%
	Taboo pay in advance	52	3%
	Limited scope of HI	28	1%
	Other reasons	13	1%

The figures above report the frequencies and percentages of respondents.

Those that do not want to join are constrained by money (32%), respond they do not need a HI scheme (15%), they have better options (28%), or that they do not trust in the government (19%). Those that would join would like to pay mostly monthly (82%), but there are 11% that would like to pay quarterly, 4% half-yearly, and 3% yearly. As explained above, 53% of those willing to join the HI scheme and go on to answer this question would accept paying 20,000 SLL per month. Forty-two percent of those who answered affirmatively to the 20,000 SLL would also accept paying 30,000 SLL. Seventy-six percent of those that are asked if they would pay 10,000 SLL for the HI scheme instead answer positively as well. Those who declared a 0 SLL willingness to pay for the HI scheme, declare, in majority, that they do not have trust in an HI scheme from the government (54%), are too poor (18%), have no trust in the government generally (15%), or think that the wealthy should pay for the scheme (12%).

#### Likelihood of answering YES to joining the health insurance scheme

S4 Table contains the results of the probit models addressing the research question of which characteristics are associated with answering YES to the HI scheme question and to paying 20,000 SLL, 30,000 SLL, or 10,000 SLL premiums for it. The coefficients in S5 Table represent the changes in the likelihood of answering YES to the question on joining the HI (Column 1); YES on the WTP the 20,000 SLL premium for the HI scheme (Column 2); YES to the 30,000 SLL premium (Column 3); and, YES to the 10,000 SLL premium (Column 4), respectively. The results show the increased or decreased likelihood of answering yes to each of those questions with respect to a base case respondent, who is a male living in the Western region, not married nor single, who is not the head of the household, working in petty trade, who has never been to school, lives in a house with a TV, who paid for the most recent medical requirement himself, and belongs to a household in poor or very poor health.

We conclude that individuals living in the Northern region are 5 percentage points more likely to answer YES to the question on whether they would join the HI scheme than those in the Western region (the omitted category), according to column 1 in S5 Table. Being in a monogamous or polygamous marriage as opposed to being divorced, separated or widowed (omitted categories) is associated with an increase in the likelihood of 3.7 and 3.8 percentage points, respectively; working as a tailor, instead of farming (omitted category) or another occupation, increases the likelihood by 2.5 and 4.6 percentage points, respectively; having had a non-formal education increases it by 4.6 percentage points; and, finally, having a TV increases it by 3 percentage points. If the respondent is female, there is a reduction in the likelihood of saying yes by 2.1 percentage points; if the respondent is a child (as opposed to the head of the household), the likelihood decreases by 4.3 percentage points; if the he or she works in the fishing industry, the likelihood is 3.3 percentage points lower; having paid for the latest medical treatment oneself decreases it by 1.8 percentage points; and, finally, if the respondent lives in a household in poor or very poor health, the likelihood of answering yes is 6.3 and 14 percentage points lower, respectively.

We also see how *different attributes are associated to increases (decreases) of the probabilities of saying yes to paying 20*,*000 SLL*, *30*,*300 SLL*, *or 10*,*000 SLL for the HI scheme* columns 2 to 4 of S5 Table. The region of origin is strongly associated with the three answers. The fact that the respondent is female only affects the likelihood of answering yes to the 30,000 SLL premium question, lowering it by 8.5 percentage points. Regarding marital status, being polygamous increases the chances of saying yes to the 20,000 SLL and the 30,000 SLL questions by 6 and 6.3 percentage points, respectively, with respect to the omitted category (divorced, separated, or widowed). Being single increases the likelihood of answering yes to the 30,000 SLL by 6.4 percentage points. With respect to the role of the respondent in the household, the coefficients for being the spouse or a child are only significant for the likelihood of answering yes to the 10,000 SLL premium, increasing it by 3 and 5.3 percentage points, respectively. In terms of occupation, being a driver is associated with an increase in answering yes to the premium of 20,000 SLL and 10,000 SLL of 9.2 and 9 percentage points, as opposed to being in petty trade. Being a biker is associated with an increase of 5.9, 5.2, and 5.9 percentage points for each one of the premiums, respectively. Being a tailor increases the odds of answering yes to the 30,000 SLL premium question by 11 percentage points. Being in farming, as opposed to petty trade, lowers the probability of answering YES to the 20,000 SLL question by 8.9 percentage points. Focusing on the effect of education, having a primary, secondary, junior, or a tertiary education increases the chances of answering yes to the 20,000 SLL premium by 10, 11, 9.6, and 12 percentage points, respectively. Education does not affect the likelihood of agreeing to the other two premiums. With respect to household amenities, having a TV results in an increase of saying yes to all three premiums questions, increasing the probabilities of saying yes to the 20,000 SLL, 30,000 SLL, and 10,000 SLL by 1, 18.4, and 7.8 percentage points, respectively.

Finally, health in the household unsurprisingly appears as a very decisive factor in explaining these likelihoods. If the households with medium health, the likelihood of answering yes to the 20,000 SLL question is lowered by 6.8 percentage points, that of saying yes to the 30,000 SLL declines by 6.7 percentage points, and that of saying yes to the 10,000 SLL by 2.7 percentage points. If the household is in poor health, the probability of answering yes to the 20,000 SLL decreases by 6.1 percentage points, and for the 10,000 SLL, by 8.2 percentage points. If the household is in very poor health, the probabilities of answering yes to the 20,000 SLL and 10,000 SLL are lowered by 10 and 6.2 percentage points, respectively.

#### Main analysis: Willingness to pay for the health insurance scheme

In this section, we present first, the aggregate WTP for the HI scheme, calculated for the full sample and estimated without controlling for the respondents’ characteristics. Second, also we obtain average WTP estimates by age groups, gender, level of education, occupation, and other characteristics, which are reported in the Supplementary Information.

The estimates in the first row of Table 6 show that the WTP for the full sample, when we evaluate the full sample at average, is 20,237.16 SLL or 3.64 USD, and is statistically significant. Given that the declared average monthly income from the business is 408,935 SLL or 74 USD, the average WTP for HI represents about 5% of the average monthly income.

**Table 6:** Aggregate willingness to pay for the HI scheme.

	WTP in SLL	WTP in USD $
Beta		
constant	20,237.16***	3.64
	(274.60)	(0.049)
Observations:	9530	9530

Standard deviations are in parentheses. The stars indicate the significance levels of the coefficients 99%, 95% and 90% as per p-value of: *** p<0.01.The exchange rate (0.000177 SLL/USD) was obtained from http://www.xe.com/currencyconverter/convert as of 11/2016.

We also estimate the average WTP for the HI scheme by *subsamples of respondents*, which may not be necessarily representative of that category given the design of the survey but are illustrative of the variation in WTP that exists in this sector of the population. We include these results in S6–S9 Tables in the supplementary information. The main conclusion of this exercise is that the WTP of the informal sector varies significantly depending on age, gender, education level, occupation, health level in the household, and difficulty they had in paying the last bill. The most extreme WTP range is wen elicited by occupation, which goes from 14,713 SLL for those in farming to 35,890 SLL for those who work as drivers. The next extreme case is by Education level: those without education have a WTP of 16,146 SLL for the HI scheme, while those who have up to tertiary education reveal a WTP up to 27,279 SLL.

#### Maximum willingness to pay for the health insurance scheme

The maximum premium that the respondents would be willing to pay (per person) for the HI Scheme: the mean is 20,766 SLL (or about 3.7 USD), the median 15,000 SLL and the standard deviation of 29,090 SLL, which indicates a markedly skewed distribution. Therefore, we estimate a Tobit model [8] using the respondents’ declared *maximum WTP for the HI scheme*—coefficients are S10 Table in the supplementary information to study which individual and household characteristics are associated with an increased (or decreased) willingness to pay for the HI scheme. We estimate two specifications of this model, where one includes the health condition of the household and the other does not. According to the Tobit model, the WTP for the HI scheme is 14,294 SLL for the base case respondent who is a single male, from the Western region, aged 31 to 45 years, who has never been to school, not working in petty trade, who lives in a home without a TV, who relied on self-treatment for the last illness in the household. The remaining covariates are continuous and so are evaluated at their sample average. With respect to the base case, there are characteristics that would make the WTP increase or decrease if changed. Living in any other region than the Western one is associated with a decrease in the maximum premium respondents are willing to pay for the HI scheme. Equally, occupation and education play a very significant role: working in farming instead of petty trade reduces the WTP, but being a driver, biker, or in another occupation category increases it. Having secondary or tertiary education as opposed to not having any also increases the WTP for the HI scheme. Finally, the number of pregnant women in the household, having a TV, and having paid oneself for the last medical episode also increase the maximum WTP for the scheme.

## Discussion

The main result of this study is that the WTP for the HI scheme elicited from using the double-bounded dichotomous choice with follow-up on a representative sample of the informal sector of Sierra Leone is 20,237 SLL, which is very close to the initial value given to the respondents in the questionnaire. The correlation of the WTP and the first bid offered to the respondents is not unexpected, as it has been proven that the findings of any double-bounded dichotomous choice model with follow up are sensitive to starting point bias. The WTP of the respondents tends to gravitate towards the initial bids that are offered, in this case 20,000 SLL. When adjusting for household characteristics, health, health care utilization, etc., the WTP rises to approximately 24,000 SLL. Despite the potential existence of this anchoring bias, we believe that the figure of 20,000 SLL is not far from the true or latent WTP for an HI scheme, because, as explained, this is also the WTP obtained by an existent study exploiting a Discrete-Choice Experiment run in 2015 on the informal sector of Sierra Leone by Joseph Kamara et al. [10]. To put our results in perspective, monthly 20,000 SLL (3.6 USD) payments for a HI scheme imply payments of about 6.8% of the 2015 World Bank [1] Sierra Leone’s GNI per capita (630 USD). The WTP of 20,000 SLL over our sample average income from business (about 408,000 SLL monthly), is about 4.8%, which is somewhat lower than if we use the GNI of the World Bank [1]. However, the 4.8% figure is above most percentages of GNI, as summarized in the systematic literature review of WTP for HI in middle- and low-income countries [5]. There are various reasons that could explain why the elicited WTP of the informal sector in Sierra Leone is higher than the literature average. The first reason is the anchoring effect, as discussed above. The second is that respondents from the informal sector have lower and more uncertain incomes than respondents from the formal. Therefore, they may be WTP higher proportions of income to avoid financial risks associated to health shocks. Finally, the devastating effect of the Ebola epidemic in Sierra Leone might explain at least partially the higher willingness to pay to ensure health coverage when the survey was done.

Our results also indicate that, depending on the region, education level, occupation, age group and other factors, the WTP varies significantly. Based on our partial analyses, the WTP for the HI scheme ranges from over 14,000 SLL to over 27,000 SLL, depending on the characteristics of the respondent and of the household he or she lives in.

**Fig 2:**
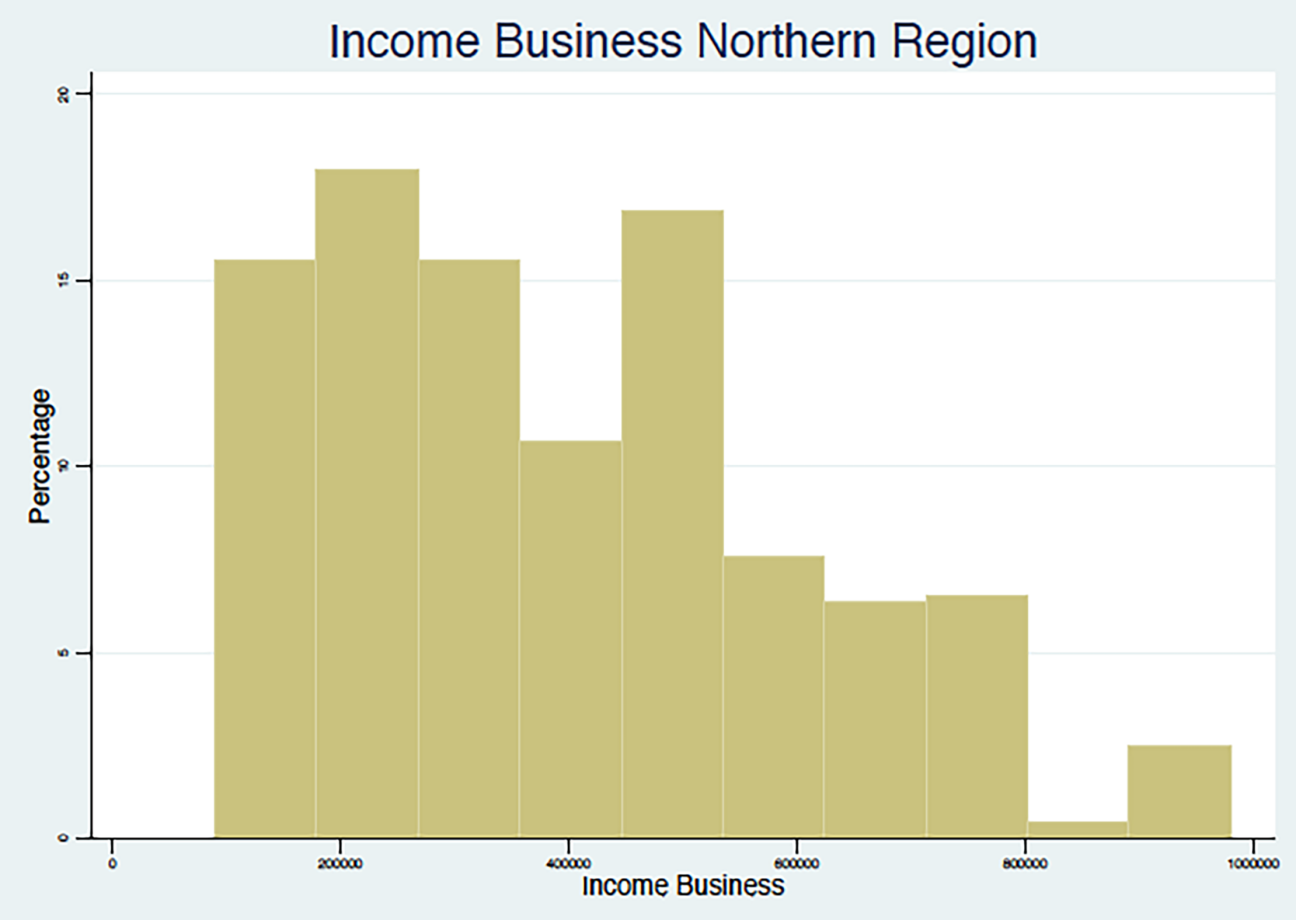
Income from Business Distribution. Northern Region.

**Fig 3:**
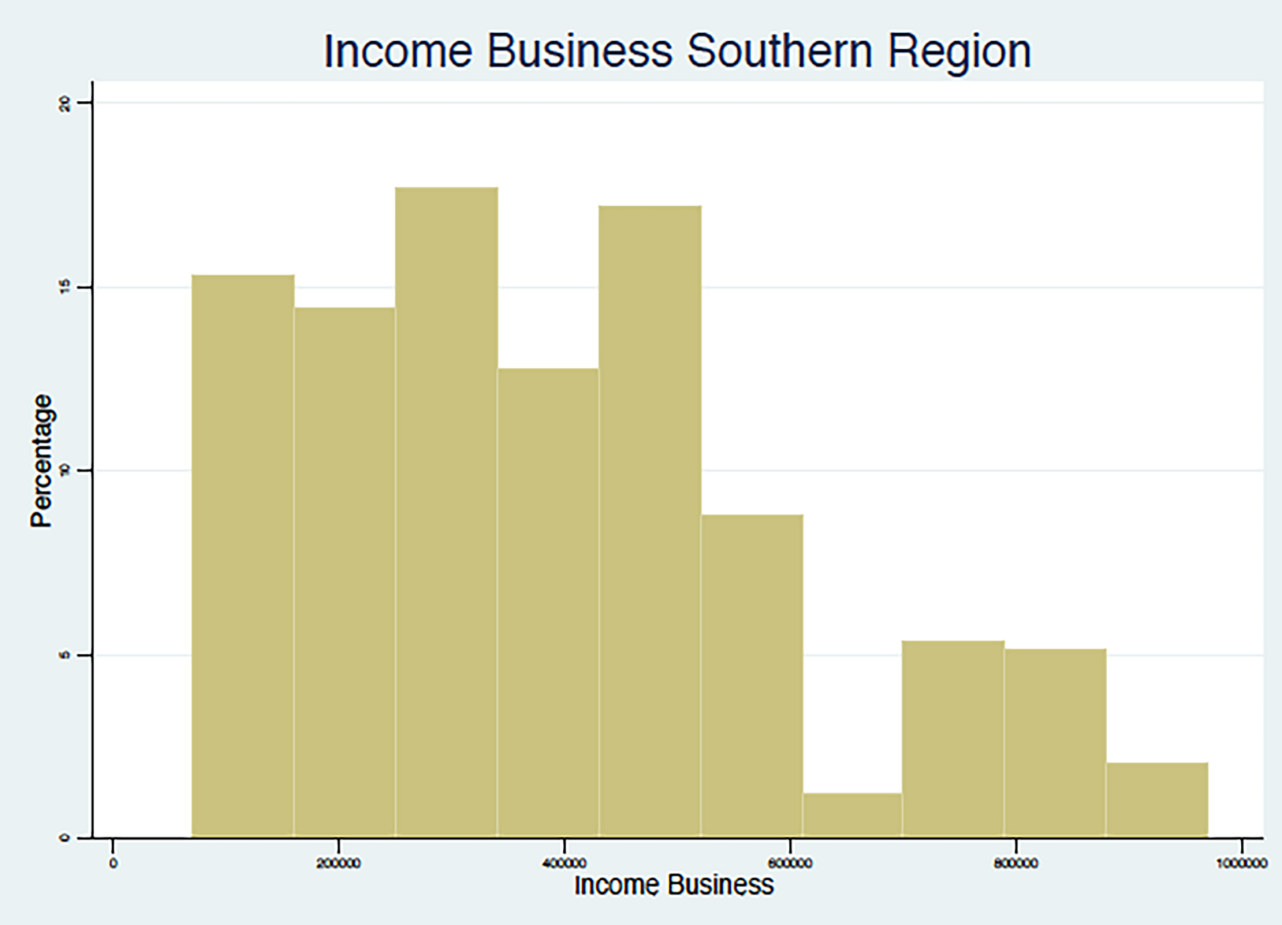
Income from Business Distribution. Southern Region.

**Fig 4:**
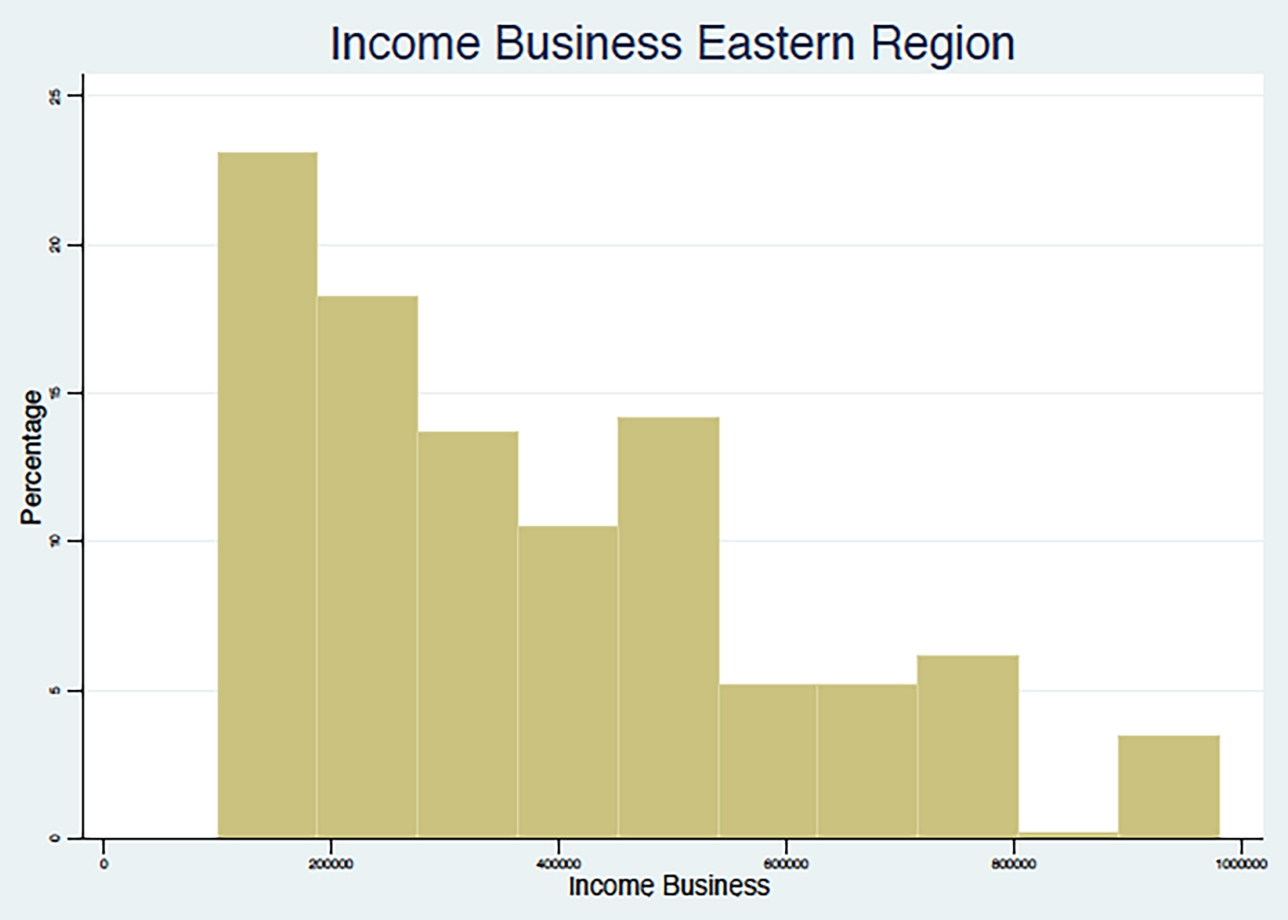
Income from Business Distribution. Eastern Region.

**Fig 5:**
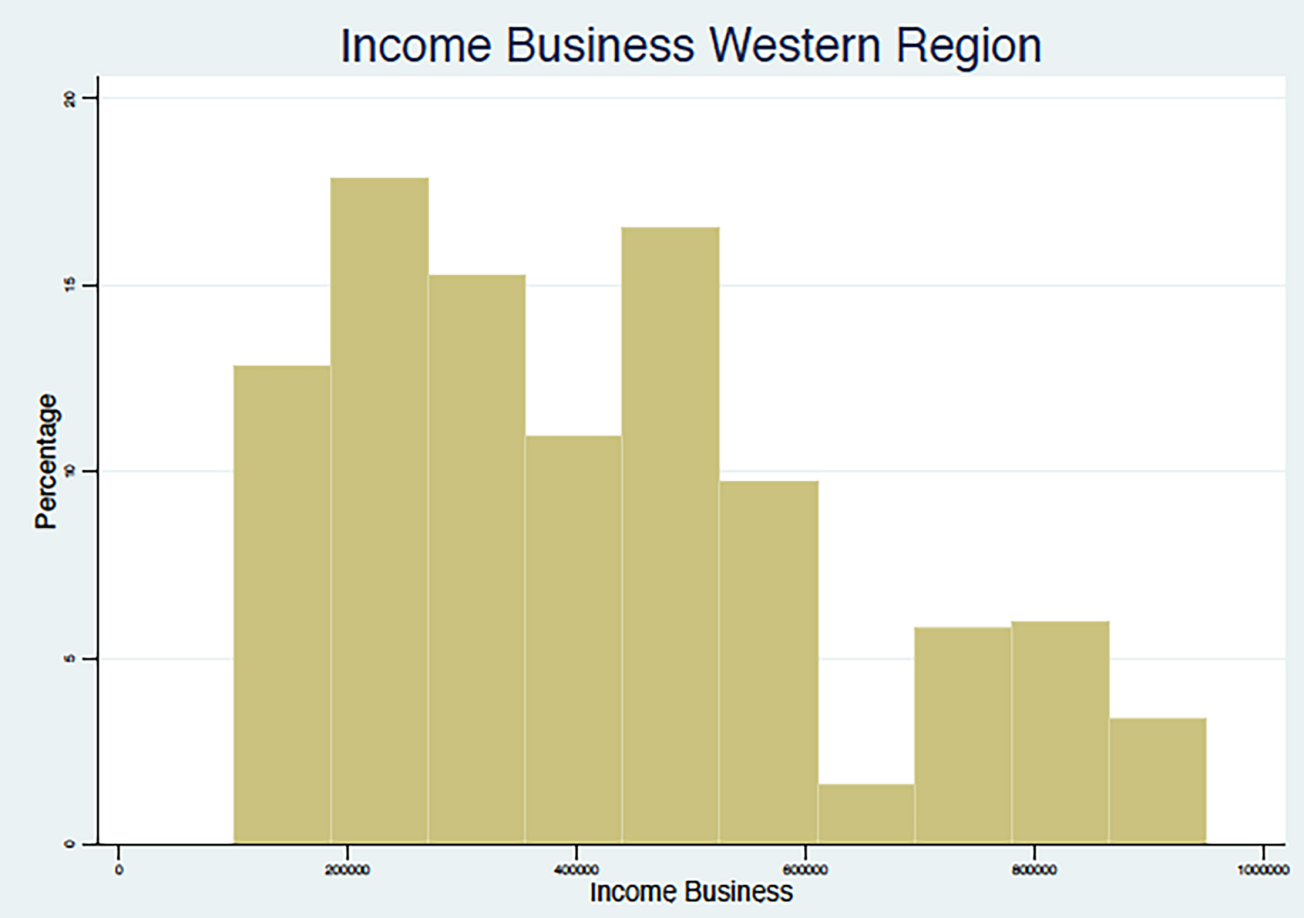
Income from Business Distribution. Western Region.

In summary, the results point to the fact that, in line with other studies in middle- and low-income countries, as summarized in Nosratnejad et al. (2016) [5], the WTP for the HI scheme unavoidably depends on the monthly income of the respondent, which appears to be associated with the region and the occupations that are most prevalent in that region, and with the education and age group of the respondent. We present below (Figs 2–5) graphical evidence of the different income distributions in each region. Note that the regions that have more mass to the right, i.e., are richer on average, are the Western and possibly the Southern.

To complete the discussion on the elicited WTP, we therefore examine the distribution of the respondents’ maximum WTP as a percentage of their declared monthly income. In the supplementary information, we present the results of mapping business income against the responses on maximum WTP for HI as a percentage of respondent income for the full sample and by region graphically (S1–S3 Figs). These diagrams illustrate some important findings of this study: First, the maximum WTP for the HI scheme when respondents are asked to state a maximum varies greatly, especially at lower income levels. Even after removing outliers, the WTP for the HI scheme varies from 0.02% to almost 30% of respondent’s income. Second, there is a negative relationship between the percentage of income that the respondents are willing to pay for the HI scheme and the magnitude of income they declare, that is, the percentage of WTP tends to decrease at higher income levels. This negative relationship may be partially due to the anchoring bias towards the 20,000 SSL premium that the respondents are offered to start the elicitation process. Finally, the patterns of the scatter plots are not identical by region. The Western and Southern regions appear to have more mass of respondents in the upper levels of income with respect to the Eastern and the Northern regions. Additionally, there are more respondents willing to pay higher percentages at higher income levels.

## Conclusions

The main implications of this study are, first, that there is a very high willingness to support the introduction of an HI package for informal care workers, with almost 90% of respondents supporting it and, second, that individuals would be willing to pay between about 14,000 SLL and about 27,000 SLL for the introduction of such a package.

The first finding of widespread support reflects what appears to be a genuine need for an HI package for the informal sector, as around two-thirds of respondents classified their health as less than good or very good. Moreover, 22% of respondent households had an individual suffering from a chronic illness and over 68% of respondents had had at least one episode of ill health in the past year, while 41% had had no treatment. Of those receiving treatment, only 53% were satisfied with it. And, of those having treatment, 58% reported difficulties with payment and that the average payment was approximately half of all their business monthly income.

In pursing the WTP for the HI scheme, of those willing to join, over 40% are WTP 30,000 SLL, while the vast majority choose the WTP level of 10,000 SLL. The univariate analysis revealed that the maximum WTP has a skewed distribution and that although its mean is 20,766 SLL, its median is only 15,000 SLL. When run the multivariate analysis of the maximum WTP controlling for covariates, the base case is approximately 14,294 SLL.

While it is difficult to disentangle the impact of different population characteristics on the WTP for the HI scheme, subsample analysis revealed that, depending on occupation, the WTP ranges from 14,713 SLL to 35,890 SLL, and depending on education, from 16,146 SLL to 27,279 SLL. Therefore, the various analyses show that a premium for the HI package could be set at approximately 20,000 SLL, but to establish a single premium for all individuals in the informal sector could be risky. The efficient functioning of an HI scheme relies on covering as much of the population as possible, in order to spread the risks and make the scheme viable. The impact of the various characteristics of the population raises the issue of how to rate premiums. In other words, setting a premium that may be too high for a big proportion of the population, could mean losing an important part of the population and have viability consequences for the operation of the scheme.

Obviously, the premium, as based on the estimated WTP, must also be linked to the HI package offered. The overall package will establish the total cost to be covered. Thus, the study highlights both the average WTP as linked to a given HI package, as well as the range of WTP as determined by the different characteristics of the informal sector population. This range of WTP will determine the availability of revenues to support the HI package. It would appear that the low end WTP estimates bottom out around 14,000 SLL, and this is consistent with many of the common characteristics of the population to be covered, while not adjusting for characteristics. Therefore, there is limited support (as this relates to common population characteristics within the informal care sector) for going beyond the 20,000 SLL-threshold.

The findings from this study open different questions that should be researched. The first one is the role of the anchor around 20,000 SLL and if the results obtained depend on this initial premium choice. Second, given the wide variation in WTP by region, education, age, and occupation, solutions that do not involve a single premium for all households should be considered. For instance, the scheme could be funded through either taxation or some sort of means tested premium, but this, of course, brings the question of how to make the informal sector individuals pay taxes that reflect their real income, which is beyond the scope of this study.

We believe that our study, despite of its limitations, makes a significant contribution to the literature for different reasons. First, there is no previous study eliciting the WTP for health insurance in Sierra Leone. Second, we apply a gold standard contingent valuation method to do so. Third, and most importantly, we believe that this study is of particular interest given the threat that the Ebola crisis revealed to the Sierra Leoneans health and livelihoods and to Global Health in general. Better access to health care at the time of beginning of this crisis would have avoided many deaths. This study is a stepping-stone towards the implementation a health insurance scheme with the aim of providing better access to health care in Sierra Leone.

## Supporting information

S1 Insert Introduction to survey. (DOCX)

S2 Insert Survey questionnaire. (DOCX)

S1 Table Survey summary statistics. Survey Sample Summary Statistics.
(DOCX)

S2 Table Health status and health care access. Summary Statistics for questions related to use of health care.
(DOCX)

S3 Table Health care services access. Summary Statistics for questions related to health care services access.
(DOCX)

S4 Table Likelihood of joining the HI scheme and responding YES to premiums of 20,000 SLL, 30,000SLL and 10,000SLL. Results of the Probit Models.
(DOCX)

S5 Table WTP for HI scheme by age group and gender. Results for WTP estimation by Age group and Gender.
(DOCX)

S6 Table WTP for HI scheme by education level. Results for WTP estimation by Education level.
(DOCX)

S7 Table WTP for HI scheme by occupation. Results for WTP estimation by Occupation.
(DOCX)

S8 Table WTP for HI scheme by household health. Results for WTP estimation by Health Status of the Household.
(DOCX)

S9 Table WTP for HI scheme by payment and having said YES to the HI scheme. Results for WTP estimation by answer to HI scheme question.
(DOCX)

S10 Table Maximum WTP for HI scheme–tobit analysis. Results of the Tobit model estimation of the Maximum WTP for the HI.
(DOCX)

S1 Fig Income and WTP for HI—all regions. (DOCX)

S2 Fig Income and WTP for HI–eastern region and Western region. (DOCX)

S3 Fig Income and WTP for HI–northern region and southern region. (DOCX)

S1 Dataset WTP for health insurance dataset. Double Bounded Dichotomous Choice Design.
(XLSX)

S1 Questionnaire WTP for health insurance. Questionnaire for WTP for Health Insurance.
(PDF)
