# Supplementary material for: Willingness to pay for health insurance in the informal sector of Sierra Leone
Source: PLoS One. 2018 May 16;13(5):e0189915. doi: 10.1371/journal.pone.0189915 (PMC5955490; doi:10.1371/journal.pone.0189915)
Supplement: S1 Insert — (DOCX) [file pone.0189915.s001.docx]

# S1 INSERT: Introduction to the Survey

## **SOCIAL HEALTH INSURANCE SCHEME SCENARIO**

***Field worker reads***: Now, I would like to ask you a few questions regarding your preferences and acceptability of health insurance. Health insurance is method used to pay for health care that helps decrease the uncertainty that may accompany the inability to pay for health care at the time it is needed. I will briefly introduce health insurance before asking you a few questions.

**Introduction to health insurance:** Health insurance is a program that pools the risk of several people in an effort to decrease the amount paid by an individual at the time health care services are needed. Ill-health occurrence is largely unpredictable for individuals; as such, the need for health-care is often highly unpredictable and very costly for most individuals. However, it is predictable for large groups. Health insurance provides an opportunity to spread the financial burden of payment over several people, thus making health care more affordable for individuals.

### **BACKGROUND**

Sickness needs to be treated immediately and waiting always causes problems. If you do not have the money available, then you will need to borrow or even sell your assets. While you run around trying to get the money together, the sick person suffers. Many times it happens that you come back with the money only to find the sick person dead. In addition, household’s out of pocket (OOP) expenditure on health care is very high. This drags many people into poverty.

In order to solve this financial problem, the Government of Sierra Leone, in its drive for universal health coverage, is planning to establish a Social Health Insurance Scheme for all Sierra Leoneans. If you register as a member of the scheme and pay your premiums, you do not need to pay again any hospital bill when you or any member of your household becomes sick.

### **BENEFITS PACKAGE**

Broadly, the scheme will cover all medical costs normally incurred at the primary health care (PHC) level. Examples of some of the costs include the following:

Doctors’ consultation fee: This is covered by the scheme when you register and pay your premiums.

Diagnostic facilities: All laboratory tests and X-ray services prescribed by the doctor or medical personnel in charge will be covered if carried out in accredited health facilities.

Inpatient stays: When you are hospitalized for a PHC illness, the scheme will cover the cost.

Surgery: The scheme will cover surgery for PHC illnesses, such as hernia, appendicitis, hydrocele, etc.

### **ENROLMENT AND PAYMENT**

You need to be paying premiums continuously for joining the scheme. CREDIT is NOT ALLOWED. After you pay the premiums, you can enjoy the benefits of the scheme after three months of registration. If you do not pay the premium, you have to pay for all services out of pocket. For instance, if you suffer from appendicitis, you have to pay the doctor’s consultation fee, inpatient stay, surgery, etc.
