# Supplementary material for: Willingness to pay for health insurance in the informal sector of Sierra Leone
Source: PLoS One. 2018 May 16;13(5):e0189915. doi: 10.1371/journal.pone.0189915 (PMC5955490; doi:10.1371/journal.pone.0189915)
Supplement: S2 Insert — (DOCX) [file pone.0189915.s002.docx]

**S2 INSERT: Survey Questionnaire**

***(Region/District/Chiefdom/00001)***

***Willingness to pay to JOIN & PAY for Social Health Insurance Scheme among Households in the Informal Sector in Sierra Leone***

Good morning/afternoon/evening! I have been contracted by the Sierra Leone Social Health Insurance (SLeSHI) Team, who are conducting a study titled “*Willingness to pay to Join and Pay for Social Health Insurance Scheme among Households in the Informal Sector in Sierra Leone,*” for the purpose of informing policies leading to the establishment of SLeSHI Scheme for all Sierra Leoneans. To conduct this study, I would like you to answer some personal questions, keeping in mind that your answers will be treated with high confidentiality and participation is voluntary. I would also like to assure you that the information that you will reveal in this interview will be solely intended for research purposes, and that your identity will not be made known to anyone.

I would like to know about your *Health Seeking Behavior, and your Willingness to Join and Pay for the proposed SLeSHI Scheme*. Do bear in mind that your honest answers to these questions will help us better understand the situation and contribute to improve the health status of all Sierra Leoneans by identifying the demand and ability to pay for the Social Health Insurance (SHI) Scheme.

**A.** **Household (Socio-demographic) Characteristics**

| **No.** | **Questions** | **Response Code**  ***(check the appropriate option or state nos.)*** | **Skip** |
| --- | --- | --- | --- |
| 100 | Respondent Sex | 1. Male 2. Female |  |
| 101 | Age of Respondent *(Years)* | 1. 18 - 30 2. 31 – 45 3. 46 – 50 4. 51 – 59 5. 60 and Above |  |
| 102 | Relation of the Respondent to the Household (HH) | 1. Head 2. Spouse 3. Child 4. Others *(parent, sibling, in-law, ward, etc):* |  |

| **No.** | **Questions** | **Response Code *(check the appropriate option or state nos.)*** | **Skip** |
| --- | --- | --- | --- |
| 103 | Respondent Marital Status | 1. Married (*Monogamous)* 2. Married *(Polygamous)* 3. Divorced 4. Widowed 5. Single 6. Separated |  |
| 104 | Occupation of the Respondent | 1. Petty Trading 2. Farming 3. Fishing 4. Tailoring 5. Commercial Bike Riding 6. Commercial Motor Driving 7. Student 8. Other________________________ |  |
| 105 | Educational Status of Respondent | 1. Primary Education 2. JSS 3. SSS 4. Tertiary 5. Non-formal 6. Never went to school |  |
| 106 | Household Size | 1. 0 – 5 yrs: Male______ Female______ 2. 6 – 18 yrs: Male______ Female______ 3. 19 – 59 yrs: Male____ Female______ 4. 60+ yrs: Male____ Female______ |  |

**.**

| **No.** | **Questions** | **Response Code *(check the appropriate option or state nos.)*** | **Skip** |
| --- | --- | --- | --- |
| 107 | How many Pregnant and Lactating Women are there in the HH? | 1. Pregnant____________ 2. Lactating____________ |  |
| 108 | Has your HH ever made use of the current Free Health Care facility? | 1. Yes 2. No | If “No” skip to Part B |
| 109 | Have they ever complained over payment of accessing the Free Health Care facility or buying of medicine? | 1. Yes 2. No |  |

**Income and Wealth Index Questions**

| **No.** | **Questions** | **Response Code *(check the appropriate option or state nos.)*** | **Skip** |
| --- | --- | --- | --- |
| 200 | Approximately how much income does your business give you? | Amount in Le__________________________ |  |
| 201 | How many of these animals do this HH own? | 1. Cows 2. Sheep 3. Goat 4. Chickens 5. Beehives 6. Others (specify type and number) ___________________ |  |
| 202 | What kind of latrine does your HH use? | 1. None 2. VIP 3. Traditional Latrine 4. Others *(specify)* |  |

| **No.** | **Questions** | **Response Code *(check the appropriate option or state nos.)*** | **Skip** |
| --- | --- | --- | --- |
| 203 | Type of roof of Respondent’s house | 1. Corrugated sheet 2. Thatch 3. Others (specify)_______________ |  |
| 204 | How many rooms are used by you and your HH for Sleeping only | Number of rooms____________________ |  |
| 205 | Do you have kitchen? | 1. Yes 2. No |  |
| 206 | Do you have separate rooms for cattle and or storage use? | 1. Yes 2. No |  |
| 207 | What is the wall of your house made of? | 1. Wooden structure 2. Mud 3. Cement 4. Other (specify)_________________ |  |
| 208 | Do you and your HH have? |  |  |
|  | 1. Functioning TV | 1. Yes 2. No |  |
|  | 1. Functioning radio | 1. Yes 2. No |  |
|  | 1. Bed and mattress | 1. Yes 2. No |  |
|  | 1. Chair and table | 1. Yes 2. No |  |
|  | 1. Car | 1. Yes 2. No |  |
|  | 1. Motor bike (okada) | 1. Yes 2. No |  |

**C. Health and Health-Related Questions**

| **No.** | **Questions** | **Response Code *(check the appropriate option or state nos.)*** | **Skip** |
| --- | --- | --- | --- |
| 300 | How do you rate the health status of your family? | 1. Very Poor 2. Poor 3. Medium 4. Good 5. Very Good |  |
| 301 | Do you or another member of the HH have chronic illness and/or disability? | 1. Yes 2. No |  |
| 302 | Have any member of the family encountered any illness during the past 3 months? | 1. Yes 2. No | If “No” skip to 304 |
| 303 | How many of the members were ill? | The number of the ill members__________ |  |
| 304 | Did you seek medical treatment for the recent episode? | 1. Yes 2. No | If “No” skip to 306 |

|  | **Questions** | **Response Code *(check the appropriate option or state nos.)*** | **Skip** |
| --- | --- | --- | --- |
| 305 | Did you get treatment? | 1. Yes 2. No | If “No” skip to 308 |
| 306 | Where did you get treatment? | 1. Self-treatment 2. Local Drug Vendor 3. Private Health Facility 4. Public Health Center 5. Public Hospital 6. Traditional Healer 7. Other (specify)___________________ |  |
| 307 | Why did you go there? | 1. The HF was physically accessible 2. The HF was not expensive 3. The HF was not too crowded 4. The health service was courteous 5. The health service was effective 6. Other (specify)___________________ |  |
| 308 | Why did you not get treatment? | 1. Consider the illness is self-limiting 2. Not enough money 3. Didn’t know anywhere to go 4. Didn’t have time to go to the HF 5. Too far to go to HF 6. Other (specify)_____________________ 7. Unknown (99) |  |

| **No.** | **Questions** | **Response Code *(check the appropriate option or state nos.)*** | **Skip** |
| --- | --- | --- | --- |
| 309 | What was the total health care cost of the HH for the treatment during the last 3 months? | Amount in Le_________________________ |  |
| 310 | Who covered the health care cost? | 1. Self 2. Family 3. Government/Free 4. Community 5. Others (specify)______________________ |  |
| 311 | How satisfied were you with the health care service and cost? | 1. Very dissatisfied 2. Dissatisfied 3. Neutral 4. Satisfied 5. Very satisfied |  |
| 312 | How did you perceive quality of the health care service in this area? | 1. Very low 2. Low 3. Neutral 4. High 5. Very high |  |
| 313 | How did you see finding money to pay for the health care? | 1. Very difficult 2. Difficult 3. Not difficult | If “No” skip to 315 |

| **No.** | **Questions** | **Response Code *(check the appropriate option or state nos.)*** | **Skip** |
| --- | --- | --- | --- |
| 314 | If paying for medical expenses was difficult, how did you get it? | 1. Drew from savings 2. Borrowing from someone 3. Assisted by relatives 4. Did extra work 5. Sell capital assets such as cows 6. Cut back on other things, food, drinks, clothes, etc 7. Others (specify)________________ |  |
| 315 | Did you borrow any money from relatives or other people to cover medical costs within the last year? | 1. Yes 2. No | If “No” skip to 317 |
| 316 | How much did you borrow? | Amount in Le_________________________ |  |
| 317 | What is the nearest conventional health institution to your home? | 1. Health center 2. Clinic (private) 3. Hospital (public) |  |
| 318 | How long does it take to reach the nearby HF from your home? | Time in minutes________________________ |  |
| 319 | What form of transportation did you use to reach the location where you obtained treatment? | 1. Personal Vehicle 2. Public Transport 3. Ambulance 4. Motor Bike (Okada) 5. Bicycle 6. Others (specify)____________________ |  |

*PLEASE EXPLAIN THE SOCIAL HEALTH INSURANCE SCHEME SCENARIO TO THE RESPONDENT BEFORE YOU ADMINISTER QUESTIONS IN SECTIONS C & D.*

**D. Willingness to Join the SLeSHI Scheme**

| **No.** | **Questions** | **Response Code *(check the appropriate option or state nos.)*** | **Skip** |
| --- | --- | --- | --- |
| 400 | Based on the scenario above, will you be willing to join SLeSHI when established? | 1. Yes 2. No | If “No” skip to 402 |
| 401 | If “Yes”, why will you join? | 1. It provides free access to medical care 2. To help others 3. For security and peace of mind in times of ill-health 4. Facing health problems frequently 5. Others (specify)_____________________ |  |
| 402 | Why will you not join the Scheme? | 1. I do not have enough money to pay 2. Do not need health insurance 3. OOP charge is better 4. Lack of trust in government programmes 5. Lack of functional HF in our area 6. No qualified medical personnel in the HF 7. Contributing money for sickness in advance is a taboo 8. Scope of illness covered by the Scheme is limited 9. Others (specify)_____________________ | Stop the interview here |

**E.** **Willingness to Pay for the SLeSHI Scheme**

| **No.** | **Questions** | **Response Code *(check the appropriate option or state nos.)*** | **Skip** |
| --- | --- | --- | --- |
| 500 | Now that you have agreed to join the SLeSHI Scheme, how frequently would you prefer to pay? | 1. Monthly 2. Quarterly 3. Half-yearly 4. Yearly 5. Others (specify)______________________ |  |
| 501 | Assuming you were to pay Le 20,000 per month per head as premium for the SLeSHI Scheme, will you be willing to pay? | 1. Yes 2. No 3. Don’t know | If either “No” or “Don’t know” skip to 503 |
| 502 | If the premium is set at Le 30,000 per month per head for the SLeSHI Scheme, would you be willing to pay? | 1. Yes 2. No 3. Don’t know | If either “No” or “Don’t know” skip to 504 |
| 503 | If the premium is set at Le 10,000 per month per head for the SLeSHI Scheme, would you be willing to pay? | 1. Yes 2. No 3. Don’t know |  |
| 504 | What is the Maximum Amount will you be willing to pay per head based on your choice mode of payment? | Amount in Le_______________________ |  |
| 505 | If the answer is Le 0.00 in 401, then tell us why is you and your household not willing to pay for the Scheme? | 1. I doubt whether it will be managed properly 2. It should be the responsibility of the government 3. Because of lack of money 4. The wealthy people should pay for the programme 5. Other (specify)_______________________ |  |
