# Supplementary material for: Willingness to pay for health insurance in the informal sector of Sierra Leone
Source: PLoS One. 2018 May 16;13(5):e0189915. doi: 10.1371/journal.pone.0189915 (PMC5955490; doi:10.1371/journal.pone.0189915)
Supplement: S1 Table — Survey Sample Summary Statistics. (DOCX) [file pone.0189915.s003.docx]

| **S1 Table: Survey Summary Statistics** | | | | |
| --- | --- | --- | --- | --- |
|  |  |  | Observations | % |
| **Region** | Region where the respondent inhabits | Eastern | 1,783 | 18% |
|  |  | Northern | 2,702 | 27% |
|  |  | Southern | 1,718 | 17% |
|  |  | Western | 3,791 | 38% |
| **Characteristics of respondent and Household:** | |  |  |  |
| **Age** | Age interval of the respondent | 18-30 years | 2,257 | 24% |
|  |  | 31-45 years | 4,182 | 44% |
|  |  | 46-50 years | 1,925 | 20% |
|  |  | 51-59 years | 796 | 8% |
|  |  | 60plus | 359 | 4% |
|  |  |  |  |  |
| **Male** | Equals 1 if male | Male | 7,175 | 72% |
|  |  |  |  |  |
|  |  |  |  |  |
| **Marital Status** | Marital status of the respondent | Married-Mon | 58 | 58% |
|  |  | Married-Pol | 1,737 | 17% |
|  |  | Divorced | 193 | 2% |
|  |  | Widowed | 690 | 7% |
|  |  | Single | 1,192 | 12% |
|  |  | Separated | 344 | 3% |
|  |  |  |  |  |
| **Occupation** | Occupation of the respondent | Petty_trade | 3,996 | 40% |
|  |  | Farming | 1,955 | 20% |
|  |  | Fishing | 467 | 5% |
|  |  | Tailor | 513 | 5% |
|  |  | Biker | 1,028 | 10% |
|  |  | Driver | 655 | 7% |
|  |  | Student | 135 | 1% |
|  |  | Other Occ | 1,239 | 12% |

|  |  |  |  |  |
| --- | --- | --- | --- | --- |
| **Education Respondent** | Highest education achieved by the respondent | Primary | 1,955 | 20% |
|  |  | Junior | 1,840 | 19% |
|  |  | Secondary | 1,570 | 16% |
|  |  | Tertiary | 461 | 5% |
|  |  | No Formal | 740 | 7% |
|  |  | None | 3,332 | 34% |

|  | |  | |  | | mean | | median | | sd | | min | | max | |
| --- | --- | --- | --- | --- | --- | --- | --- | --- | --- | --- | --- | --- | --- | --- | --- |
| **Income from business** | | Income from the business (SLL) | | 9,524 | | 408,935 | | 400,000 | | 270,007 | | 0 | | 10,000,000 | |

|  |  |  | Observations | % |
| --- | --- | --- | --- | --- |
| **House Material** | Material the walls of the house are made of: | Wood | 426 | 4% |
|  |  | Mud | 5,688 | 58% |
|  |  | Cement | 3,557 | 36% |
|  |  | Other | 188 | 2% |
|  |  |  |  |  |
|  |  | Observations | % | |
| **Items in the house** | Kitchen | 9,907 | 73% |  |
|  | Stable | 9,581 | 43% |  |
|  | TV | 9,905 | 87% |  |
|  | Radio | 9,934 | 96% |  |
|  | Mattress | 9,932 | 85% |  |
|  | Chair and Table | 9,865 | 10% |  |
|  | Car | 9,879 | 21% |  |
|  |  |  |  |  |
|  |  | Observations | mean | sd |
| **Household Size** | Number of members | 9,994 | 6.09 | 3.85 |
|  |  |  |  |  |
| **Pregnant and lactating women** | Number of pregnant women | 9,994 | 0.22 | 0.55 |
|  | Number of lactating women | 9,994 | 0.49 | 0.77 |

| **HH number of rooms** | Number of rooms | 9,378 | 3.31 | 1.84 | 1 | 48 |
| --- | --- | --- | --- | --- | --- | --- |
|  |  |  |  |  |  |  |
|  |  |  | Percentage |  | min | max |
| **Number of Animals in HH** | cows | 9,994 | 6% |  | 0 | 25 |
|  | sheep | 9,994 | 43% |  | 0 | 40 |
|  | goats | 9,994 | 72% |  | 0 | 22 |
|  | chickens | 9,994 | 271% |  | 0 | 99 |
|  | beehives | 9,994 | 7% |  | 0 | 20 |
