# Supplementary material for: Willingness to pay for health insurance in the informal sector of Sierra Leone
Source: PLoS One. 2018 May 16;13(5):e0189915. doi: 10.1371/journal.pone.0189915 (PMC5955490; doi:10.1371/journal.pone.0189915)
Supplement: S2 Table — Summary Statistics for questions related to use of health care. (DOCX) [file pone.0189915.s004.docx]

| **S2 Table: Health Status and Health Care Access** | | | |  | | |
| --- | --- | --- | --- | --- | --- | --- |
| **Health Status HH** |  | Very Poor | 502 | | 5% |  |
|  |  | Poor | 1,958 | | 20% |  |
|  |  | Med | 4,299 | | 43% |  |
|  |  | Good | 2,864 | | 29% |  |
|  |  | Very Good | 341 | | 3% |  |
|  |  |  |  | |  |  |
| **Chronic Disease/Disability in HH** |  | Yes | 2,092 | | 22% |  |
|  |  | No | 7,330 | | 77% |  |
|  |  |  |  | |  |  |
| **Illness last three months** |  | Yes | 6,337 | | 68% |  |
|  |  | No | 3,017 | | 32% |  |
|  |  |  |  | |  |  |
| **Where got treatment** |  | HCT Self | 1,157 | | 15% |  |
|  |  | Lcal Drug Vendor | 1,352 | | 17% |  |
|  |  | Private HCFac | 446 | | 6% |  |
|  |  | Public HCentre | 2,435 | | 31% |  |
|  |  | Public Hospital | 2,351 | | 30% |  |
|  |  | Traditional Healther | 205 | | 3% |  |
|  |  | OtherHC | 21 | | 0% |  |
|  |  |  |  | |  |  |
| **Why treatment there** |  | AccessHC | 3,227 | | 49% |  |
|  |  | CheapHC | 1,795 | | 27% |  |
|  |  | crowdedHC | 169 | | 3% |  |
|  |  | CourteousHC | 202 | | 3% |  |
|  |  | EffectiveHC | 1,092 | | 17% |  |
|  |  | OtherRHC | 131 | | 2% |  |

|  |  |  |  |  |
| --- | --- | --- | --- | --- |
| **Why did you not get treatment** |  | SelflimitIll | 828 | 41% |
|  |  | Not Money | 638 | 32% |
|  |  | Not Know | 46 | 2% |
|  |  | No Time | 100 | 5% |
|  |  | Too Far | 254 | 13% |
|  |  | Other | 123 | 6% |
|  |  | Unknown | 22 | 1% |
|  |  |  |  |  |
| **How satisfied were you with cost and treatment?** |  | Very dissatisfied | 570 | 7% |
|  |  | Disat-HC | 1,704 | 21% |
|  |  | Neutral-HC | 1,451 | 18% |
|  |  | Sat-HC | 3,884 | 48% |
|  |  | Very Satisfied | 519 | 6% |
|  |  |  |  |  |
| **Quality of HCS in this area?** |  | Very Low | 961 | 10% |
|  |  | Low | 2,915 | 31% |
|  |  | Neutral | 2,980 | 32% |
|  |  | High | 2,100 | 23% |
|  |  | Very High | 338 | 4% |
|  |  |  |  |  |
| **Difficulty finding money to pay for HC?** |  | Very Difficult | 3,559 | 38% |
|  |  | Difficult | 4,973 | 53% |
|  |  | Not Difficult | 781 | 8% |
|  |  |  |  |  |
| **If it was expensive, how did you pay?** |  | Savings | 1,904 | 40% |
|  |  | Borrow | 903 | 19% |
|  |  | Relatives | 740 | 16% |
|  |  | Extra work | 755 | 16% |
|  |  | Sell Assets | 86 | 2% |
|  |  | Cut down other expenses | 279 | 6% |
|  |  | Other Sources | 41 | 1% |
|  |  |  |  |  |
| **Did you borrow money from relatives or other people to cover medical expenses** |  | No | 6,022 | 69% |
|  |  | Yes | 2,704 | 31% |

|  |  |  |  | mean | sd | Min | Max |
| --- | --- | --- | --- | --- | --- | --- | --- |
| **How much did you pay in total?** |  | Amount | 7,380 | 165,576.7 | 286,347 | 0 | 1,600,000 |

|  |  |  |  | % | Avge(Le) | USD |
| --- | --- | --- | --- | --- | --- | --- |
| **Who covered health care cost?** |  | SelfPayHC | 6,102 | 82% | 166,042.8 | $29.89 |
|  |  | FamilyHC | 1,084 | 15% | 185,704.3 | $33.43 |
|  |  | GovntHC | 135 | 2% | 114,513.3 | $20.61 |
|  |  | CommunityHC | 94 | 1% | 156,904.3 | $28.24 |
|  |  | OtherSourHC | 58 | 1% | 77,741.4 | $13.99 |
