# Supplementary material for: Willingness to pay for health insurance in the informal sector of Sierra Leone
Source: PLoS One. 2018 May 16;13(5):e0189915. doi: 10.1371/journal.pone.0189915 (PMC5955490; doi:10.1371/journal.pone.0189915)
Supplement: S3 Table — Summary Statistics for questions related to health care services access. (DOCX) [file pone.0189915.s005.docx]

**S3 Table: Health Care Services Access**

| **Nearest conventional health centre?** | |  | | Health Centre | | | 4,031 | | 42% | |
| --- | --- | --- | --- | --- | --- | --- | --- | --- | --- | --- |
|  | |  | | Private Clinic | | | 1,341 | | 14% | |
|  | |  | | Hospital | | | 4,323 | | 45% | |
|  | |  | |  | | |  | |  | |
| **How did you reach the HC?** | |  | | Vehicle | | | 313 | | 3% | |
|  | |  | | Public Transport | | | 1,631 | | 17% | |
|  | |  | | Ambulance | | | 42 | | 0.5% | |
|  | |  | | Moto | | | 4,930 | | 53% | |
|  | |  | | Bike | | | 89 | | 1% | |
|  | |  | | Other Transport | | | 2,376 | | 25% | |
|  | |  | |  | |  | mean | | sd | |
| **Time invested in travelling to HC** | | Number of minutes | | | |  | 25.18 | | 286,691 | |

| **How often would you like to pay?** |  | Monthly | 7,108 | 82% |
| --- | --- | --- | --- | --- |
|  |  | Quarterly | 919 | 11% |
|  |  | Half-yearly | 318 | 4% |
|  |  | Yearly | 260 | 3% |
|  |  | OtherF | 41 | 0% |
|  |  |  |  |  |
| **If the premium was 20,000/month would you join?** |  | Yes | 4,492 | 53% |
|  |  | No | 3,828 | 45% |
|  |  | Don't know | 147 | 2% |
|  |  |  |  |  |
|  |  |  |  |  |
| **If you said yes to 20,000/month, would you pay 30,000/month?** |  | Yes | 2,083 | 42% |
|  |  | No | 2,601 | 53% |
|  |  | Don't know | 249 | 5% |
|  |  |  |  |  |
| **If you said NO to 20,000/month, would you pay 10,000/month?** |  | Yes | 5,958 | 76% |
|  |  | No | 1,759 | 23% |
|  |  | Don't know | 82 | 1% |
|  |  |  |  |  |
| **If your maximum premium is SLL 0, why?** |  | No trust in management | 47 | 15% |
|  |  | No trust in HI government | 168 | 54% |
|  |  | Too poor | 55 | 18% |
|  |  | Wealthy, can pay | 38 | 12% |
|  |  | Other reasons | 6 | 2% |
