# Supplementary material for: Willingness to pay for health insurance in the informal sector of Sierra Leone
Source: PLoS One. 2018 May 16;13(5):e0189915. doi: 10.1371/journal.pone.0189915 (PMC5955490; doi:10.1371/journal.pone.0189915)
Supplement: S4 Table — Results of the Probit Models. (DOCX) [file pone.0189915.s006.docx]

**S4 Table: Likelihood of joining the HI Scheme and**

**Responding YES to premiums of 20,000 SLL, 30,000SLL and 10,000SLL**

|  | (1) | | (2) | | (3) | | (4) |
| --- | --- | --- | --- | --- | --- | --- | --- |
|  | Likelihood to Join HI | | Yes20000 | | Yes30000 | | Yes10000 |
| Eastern (d) | -0.009 | | -0.044** | | 0.035 | | -0.043*** |
|  | (0.009) | | (0.018) | | (0.023) | | (0.016) |
| Northern (d) | 0.050*** | | -0.076*** | | -0.089*** | | -0.064*** |
|  | (0.008) | | (0.017) | | (0.021) | | (0.015) |
| Southern (d) | 0.002 | | -0.127*** | | -0.119*** | | -0.031* |
|  | (0.009) | | (0.018) | | (0.023) | | (0.017) |
| Female (d) | -0.021** | | 0.006 | | -0.085*** | | 0.004 |
|  | (0.010) | | (0.018) | | (0.024) | | (0.015) |
| Monogamous (d) | 0.037*** | | 0.008 | | 0.044 | | 0.027 |
|  | (0.011) | | (0.021) | | (0.028) | | (0.018) |
| Poligamous (d) | 0.038*** | | 0.060** | | 0.063* | | 0.024 |
|  | (0.010) | | (0.024) | | (0.032) | | (0.020) |
| Single (d) | -0.001 | | -0.031 | | 0.064* | | 0.009 |
|  | (0.014) | | (0.030) | | (0.037) | | (0.024) |
| Spouse (d) | -0.013 | | -0.032 | | 0.023 | | 0.030* |
|  | (0.011) | | (0.020) | | (0.027) | | (0.016) |
| Child (d) | -0.043** | | -0.033 | | -0.018 | | 0.053** |
|  | (0.021) | | (0.035) | | (0.046) | | (0.026) |
| Fishing (d) | -0.033* | | -0.041 | | -0.045 | | 0.028 |
|  | (0.019) | | (0.032) | | (0.042) | | (0.026) |
| Farming (d) | -0.004 | | -0.089*** | | -0.019 | | -0.022 |
|  | (0.010) | | (0.018) | | (0.025) | | (0.015) |
| Tailor (d) | 0.025* | | 0.020 | | 0.110*** | | 0.022 |
|  | (0.014) | | (0.028) | | (0.035) | | (0.022) |
| Biker (d) | -0.002 | | 0.059*** | | 0.052* | | 0.059*** |
|  | (0.012) | | (0.022) | | (0.027) | | (0.017) |
| Driver (d) | -0.015 | | 0.092*** | | 0.003 | | 0.090*** |
|  | (0.016) | | (0.027) | | (0.033) | | (0.019) |
| OtherOcc (d) | 0.028*** | | -0.017 | | -0.037 | | 0.027 |
|  | (0.010) | | (0.021) | | (0.026) | | (0.017) |
| NonFormalEd (d) | 0.046*** | | 0.094*** | | 0.044 | | 0.008 |
|  | (0.011) | | (0.023) | | (0.032) | | (0.021) |
| Primary (d) | -0.000 | | 0.105*** | | 0.021 | | -0.024 |
|  | (0.010) | | (0.017) | | (0.024) | | (0.016) |
| Junior (d) | -0.003 | | 0.110*** | | -0.011 | | 0.004 |
|  | (0.010) | | (0.018) | | (0.024) | | (0.016) |
| Secondary (d) | -0.015 | | 0.096*** | | 0.040 | | 0.004 |
|  | (0.012) | | (0.020) | | (0.026) | | (0.018) |
| Tertiary (d) | 0.006 | | 0.120*** | | 0.027 | | 0.029 |
|  | (0.016) | | (0.028) | | (0.038) | | (0.025) |
| Tv | 0.030*** | | 0.188*** | | 0.184*** | | 0.078*** |
|  | (0.011) | | (0.019) | | (0.029) | | (0.018) |
| SelfPayHC (d) | -0.018** | | 0.011 | | 0.043** | | 0.014 |
|  | (0.008) | | (0.016) | | (0.021) | | (0.014) |
| HealthMed (d) | -0.019** | | -0.068*** | | -0.067*** | | -0.027** |
|  | (0.009) | | (0.015) | | (0.019) | | (0.013) |
| HealthPoor (d) | -0.063*** | | -0.061*** | | -0.016 | | -0.082*** |
|  | (0.012) | | (0.018) | | (0.023) | | (0.017) |
| HealthVPoor (d) | -0.140*** | | -0.101*** | | -0.028 | | -0.062** |
|  | (0.023) | | (0.030) | | (0.039) | | (0.039) |
| Observations | 7819 | | 6685 | | 4041 | | 4041 |
| Marginal effects. |  |  | |  | |  | |
| Note: Robust Standard Errors in Parentheses. Each column has different number of observations because only a subset of respondents that answer they are willing to pay for the insurance scheme is asked if they would be willing to pay a premium of 20,000 SLL. And only subsets of those answering the 20,000 SLL premium question go on to answer the other two questions. | | | | | | | |
