# Supplementary material for: Willingness to pay for health insurance in the informal sector of Sierra Leone
Source: PLoS One. 2018 May 16;13(5):e0189915. doi: 10.1371/journal.pone.0189915 (PMC5955490; doi:10.1371/journal.pone.0189915)
Supplement: S5 Table — Results for WTP estimation by Age group and Gender. (DOCX) [file pone.0189915.s007.docx]

**S5 Table: WTP for HI Scheme by Age Group and Gender**

|  | (1) | (2) | (3) | (4) | (5) | (6) | (7) | (8) | (10) |
| --- | --- | --- | --- | --- | --- | --- | --- | --- | --- |
|  | Monogamous Male | Polygamous Male | Single Male | Age 31–45 | Age 46–59 | Age 51–59 | Age 60+ | Male | Female |
| WTP | 21,613.41*** | 23,302.70*** | 19,047.89*** | 21,234.39*** | 21,458.28*** | 20,390.95*** | 14,950.18*** | 21,396.32*** | 17,315.29*** |
|  | (408.13) | (915.46) | (778.58) | (435.42) | (685.71) | (1014.17) | (1523.05) | (326.27) | (508.71) |
| USD | 3.89 | 4.19 | 3.43 | 3.82 | 3.86 | 3.67 | 2.69 | 3.85 | 3.12 |
| Obs. | 4,155 | 1,378 | 856 | 4,182 | 1,925 | 796 | 359 | 6,850 | 2,659 |

Standard deviations are in parentheses. . The stars indicate the significance levels of the coefficients 99%, 95% and 90% as per p-value of : *** p<0.01, ** p<0.05, p<0.1. Same exchange rate used as for Table 7.
