# Supplementary material for: Willingness to pay for health insurance in the informal sector of Sierra Leone
Source: PLoS One. 2018 May 16;13(5):e0189915. doi: 10.1371/journal.pone.0189915 (PMC5955490; doi:10.1371/journal.pone.0189915)
Supplement: S6 Table — Results for WTP estimation by Education level. (DOCX) [file pone.0189915.s008.docx]

**S6 Table: WTP for HI Scheme by Education Level**

|  | (1) | (2) | (3) | (4) | (5) |
| --- | --- | --- | --- | --- | --- |
| Education | Primary | Secondary | Tertiary | Non-formal | None |
| WTP | 21,607.21*** | 21,692.42*** | 27,279.32*** | 24,277.51*** | 16,146.51*** |
|  | (699.92) | (693.19) | (1,307.37) | (973.93) | (430.69) |
| USD | 3.89 | 3.90 | 4.91 | 4.37 | 2.91 |
| Observations | 1,831 | 1,517 | 447 | 689 | 3,248 |

Standard deviations are in parentheses. . The stars indicate the significance levels of the coefficients 99%, 95% and 90% as per p-value of: *** p<0.01, ** p<0.05, p<0.1. Same exchange rate used as for Table 7.
