# Supplementary material for: Willingness to pay for health insurance in the informal sector of Sierra Leone
Source: PLoS One. 2018 May 16;13(5):e0189915. doi: 10.1371/journal.pone.0189915 (PMC5955490; doi:10.1371/journal.pone.0189915)
Supplement: S7 Table — Results for WTP estimation by Occupation. (DOCX) [file pone.0189915.s009.docx]

|  | (1) | (2) | (3) | (4) | (5) | (6) |
| --- | --- | --- | --- | --- | --- | --- |
| Occupation | Farming | Fishing | Tailor | Biker | Driver | Others |
| WTP | 14,713.11*** | 19,383.88*** | 23,532.88*** | 23,260.83*** | 35,890.02*** | 23,704.19*** |
|  | (580.49) | (915.22) | (1,460.00) | (805.93) | (1,776.44) | (716.55) |
| USD | 2.65 | 3.49 | 4.24 | $4.19 | $6.46 | $4.27 |
| Observations | 1887 | 461 | 469 | 969 | 646 | 1221 |

**S7 Table: WTP for HI Scheme by Occupation**

Standard deviations are in parentheses. . The stars indicate the significance levels of the coefficients 99%, 95% and 90% as per p-value of: *** p<0.01, ** p<0.05, p<0.1. Same exchange rate used as for Table 7.
