# Supplementary material for: Willingness to pay for health insurance in the informal sector of Sierra Leone
Source: PLoS One. 2018 May 16;13(5):e0189915. doi: 10.1371/journal.pone.0189915 (PMC5955490; doi:10.1371/journal.pone.0189915)
Supplement: S8 Table — Results for WTP estimation by Health Status of the Household. (DOCX) [file pone.0189915.s010.docx]

**S8 Table: WTP for HI Scheme by Household Health**

|  | (1) | (2) | (3) | (4) | (5) |
| --- | --- | --- | --- | --- | --- |
| Health | Chronic | Ill 3 months | Very Poor | Medium | Good |
| WTP | 20,368.50*** | 20,988.21*** | 17,004.83*** | 19,124.19*** | 24,661.53*** |
|  | (661.19) | (330.03) | (615.16) | (363.67) | (553.44) |
| USD | 3.66 | 3.77 | 3.06 | 3.44 | 4.43 |
| Observations | 2,092 | 6,337 | 2,354 | 4,053 | 3,093 |

Standard deviations are in parentheses. . The stars indicate the significance levels of the coefficients 99%, 95% and 90% as per p-value of: *** p<0.01, ** p<0.05, p<0.1. Same exchange rate used as for Table 7.
