# Supplementary material for: Willingness to pay for health insurance in the informal sector of Sierra Leone
Source: PLoS One. 2018 May 16;13(5):e0189915. doi: 10.1371/journal.pone.0189915 (PMC5955490; doi:10.1371/journal.pone.0189915)
Supplement: S9 Table — Results for WTP estimation by answer to HI scheme question. (DOCX) [file pone.0189915.s011.docx]

**S9 Table: WTP for HI Scheme by Payment and having said yes to HI Scheme**

|  | (1) | (2) | (3) | (4) | (5) |
| --- | --- | --- | --- | --- | --- |
| Payment | Self-Pay | Family | Different | Borrow | Yes to HI |
| WTP | 24,661.53*** | 19,407.30*** | 20,812.81*** | 24,426.29*** | 23,656.26*** |
|  | (553.44) | (886.46) | (291.98) | (962.71) | (262.99) |
| USD | 4.44 | 3.49 | 3.75 | 4.40 | 4.26 |
| Observations | 3,093 | 1,072 | 8,150 | 875 | 8,409 |

Standard deviations are in parentheses. . The stars indicate the significance levels of the coefficients 99%, 95% and 90% as per p-value of : *** p<0.01, ** p<0.05, p<0.1. Same exchange rate used as for Table 7.
