# Supplementary material for: Willingness to pay for health insurance in the informal sector of Sierra Leone
Source: PLoS One. 2018 May 16;13(5):e0189915. doi: 10.1371/journal.pone.0189915 (PMC5955490; doi:10.1371/journal.pone.0189915)
Supplement: S10 Table — Results of the Tobit model estimation of the Maximum WTP for the HI. (DOCX) [file pone.0189915.s012.docx]

**S10 Table: Maximum WTP for HI scheme – Tobit Analysis**

|  | (1) | (2) | | |
| --- | --- | --- | --- | --- |
|  | MaxHILe | MaxHILe | | |
| Eastern | -1730.6 | -1990.9* | |  |
|  | (993.3) | (988.5) | |  |
| Northern | -2212.7* | -2544.6** | |  |
|  | (935.3) | (931.7) | |  |
| Southern | -4263.9*** | -4236.6*** | |  |
|  | (1017.7) | (1015.6) | |  |
| Female | 1246.3 | 1195.3 | |  |
|  | (958.0) | (958.5) | |  |
| Head | -776.3 | -706.9 | |  |
|  | (962.2) | (961.7) | |  |
| ag1830 | -4359.4*** | -4356.6*** | |  |
|  | (982.5) | (982.3) | |  |
| ag4650 | 1183.4 | 1183.9 | |  |
|  | (923.4) | (923.6) | |  |
| ag5159 | -855.2 | -927.5 | |  |
|  | (1344.3) | (1345.1) | |  |
| ag60plus | -1827.5 | -1913.1 | |  |
|  | (1935.5) | (1936.0) | |  |
| Single | 2818.4* | 2851.3* | |  |
|  | (1279.9) | (1278.3) | |  |
| NonFormalEd | 1758.4 | 1779.2 | |  |
|  | (1372.0) | (1371.8) | |  |
| Primary | 1365.9 | 1358.1 | |  |
|  | (1004.3) | (1004.0) | |  |
| Junior | 242.3 | 223.8 | |  |
|  | (1051.4) | (1051.1) | |  |
| Secondary | 3089.9** | 3145.7** | |  |
|  | (1171.7) | (1170.8) | |  |
| Tertiary | 4511.7** | 4477.0** | |  |
|  | (1720.3) | (1721.5) | |  |
| sizeHH | 144.0 | 167.4 | |  |
|  | (103.0) | (102.9) | |  |
| pregnant | 3842.9*** | 3913.6*** | |  |
|  | (645.4) | (642.8) | |  |
| zero5MALE | 249.8 | 274.2 | |  |
|  | (460.0) | (457.3) | |  |
| Fishing | -1323.6 | -1101.9 | |  |
|  | (1783.9) | (1784.4) | |  |
| Farming | -4162.8*** | -4091.3*** | |  |
|  | (998.5) | (997.3) | |  |
| Tailor | 498.1 | 421.5 | |  |
|  | (1568.0) | (1568.7) | |  |
| Biker | 3968.6** | 4022.9** | |  |
|  | (1265.9) | (1265.0) | |  |
| Driver | 6342.5*** | 6664.5*** | |  |
|  | (1570.7) | (1568.3) | |  |
| OtherOcc | 2744.5* | 2875.4* | |  |
|  | (1177.2) | (1176.8) | |  |
| tv | 4842.5*** | 4790.9*** | |  |
|  | (1091.1) | (1090.8) | |  |
| timefinal2 | -0.389 | -0.545 | |  |
|  | (5.447) | (5.429) | |  |
| SelfPayHC | 1982.3* | 1949.2* | |  |
|  | (892.5) | (892.1) | |  |
| HealthMed | -2264.7** |  | |  |
|  | (840.3) |  | |  |
| HealthPoor | -517.1 |  | |  |
|  | (1013.3) |  | |  |
| HealthVPoor | 3969.0* |  | |  |
|  | (1657.2) |  | |  |
| _cons | 15296.3*** | 14294.9*** | |  |
|  | (1915.1) | (1822.8) | |  |
| sigma |  |  | |  |
| _cons | 28338.6*** | 28364.3*** | |  |
|  | (243.5) | (243.5) | |  |
| Observations | 6786 | 6795 | |  |
| Note: Robust Standard Errors in Parentheses. The stars indicate the significance levels of the coefficients 99%, 95% and 90% as per p-value of : *** p<0.01, ** p<0.05, p<0.1.  Colum (1) controls for health of household. | | |  |  |
