# Supplementary material for: Willingness to pay for health insurance in the informal sector of Sierra Leone
Source: PLoS One. 2018 May 16;13(5):e0189915. doi: 10.1371/journal.pone.0189915 (PMC5955490; doi:10.1371/journal.pone.0189915)
Supplement: S2 Fig — (DOCX) [file pone.0189915.s014.docx]

**S2 Figure A and B: Income and WTP for HI for Eastern and Western Regions**
