# Supplementary material for: Willingness to pay for health insurance in the informal sector of Sierra Leone
Source: PLoS One. 2018 May 16;13(5):e0189915. doi: 10.1371/journal.pone.0189915 (PMC5955490; doi:10.1371/journal.pone.0189915)
Supplement: S1 Questionnaire — Questionnaire for WTP for Health Insurance. (PDF) [file pone.0189915.s017.pdf]

(Region/District/Chiefdom/00001)

SURVEY QUESTIONNAIRE

Willingness to pay to JOIN & PAY for Social Health Insurance Scheme among Households in the Informal Sector in Sierra Leone

Good morning/afternoon/evening! I have been contracted by the Sierra Leone Social Health Insurance (SLeSHI) Team, who are conducting a study titled; "*Willingness to pay to Join and Pay for Social Health Insurance Scheme among Households in the Informal Sector in Sierra Leone*", for the purpose of informing policies leading to the establishment of SLeSHI Scheme for ALL Sierra Leoneans. To conduct this study, I would like you to answer some personal questions and your answers will be treated with high confidentiality and participation is voluntary. I would also like to assure you that the information that you will reveal in this interview will be solely for purposes of research, and that your identity will not be made known to any.

I would like to know about your *Health Seeking Behaviour, and your Willingness to Join and Pay for the proposed SLeSHI Scheme*. Do bear in mind that your honest answer to these questions will help us to better understand the situation and will contribute to improve the health status of ALL Sierra Leoneans by identifying the demand and ability to pay for Social Health Insurance (SHI) Scheme.

A. Household (Socio-demographic) Characteristics

No.	Questions	Response Code<br>*(check the appropriate option or state nos.)*	Skip
100	Respondent Sex	1. Male ☐<br>2. Female ☐	
101	Age of Respondent *(Years)*	1. 18 - 30 ☐<br>2. 31 – 45 ☐<br>3. 46 – 50 ☐<br>4. 51 – 59 ☐<br>5. 60 and Above ☐	
102	Relation of the Respondent to the Household (HH)	1. Head ☐<br>2. Spouse ☐<br>3. Child ☐<br>4. Others *(parent, sibling, in-law, ward, etc):* ☐	

No.	Questions	Response Code *(check the appropriate option or state nos.)*	Skip
103	Respondent Marital Status	1. Married (*Monogamous)* ☐<br>2. Married *(Polygamous)* ☐<br>3. Divorced ☐<br>4. Widowed ☐<br>5. Single ☐<br>6. Separated ☐	
104	Occupation of the Respondent	1. Petty Trading ☐<br>2. Farming ☐<br>3. Fishing ☐<br>4. Tailoring ☐<br>5. Commercial Bike Riding ☐<br>6. Commercial Motor Driving ☐<br>7. Student ☐<br>8. Other______________________ ☐	
105	Educational Status of Respondent	1. Primary Education ☐<br>2. JSS ☐<br>3. SSS ☐<br>4. Tertiary ☐<br>5. Non-formal ☐<br>6. Never went to school ☐	
106	Household Size	1. 0 – 5 yrs: Male_____ Female_____<br>2. 6 – 18 yrs: Male_____ Female_____<br>3. 19 – 59 yrs: Male___ Female_____<br>4. 60+ yrs: Male___ Female______	

No.	Questions	Response Code *(check the appropriate option or state nos.)*	Skip
107	How many Pregnant and Lactating Women are there in the HH?	1. Pregnant___________<br>2. Lactating___________	
108	Has your HH ever made use of the current Free Health Care facility?	1. Yes ☐<br>2. No ☐	If "No" skip to Part B
109	Have they ever complained over payment of accessing the Free Health Care facility or buying of medicine?	1. Yes ☐<br>2. No ☐	

B. Income and Wealth Index Questions

No.	Questions	Response Code *(check the appropriate option or state nos.)*	Skip
200	Approximately how much income does your business give you?	Amount in Le________________________	
201	How many of these animals do this HH own?	1. Cows ☐<br>2. Sheep ☐<br>3. Goat ☐<br>4. Chickens ☐<br>5. Beehives ☐<br>6. Others (specify type and number) _________________ ☐	

202	What kind of latrine does your HH use?	1. None ☐<br>2. VIP ☐<br>3. Traditional Latrine ☐<br>4. Others *(specify)*	

No.	Questions	Response Code *(check the appropriate option or state nos.)*	Skip
203	Type of roof of Respondent's house	1. Corrugated sheet ☐<br>2. Thatch ☐<br>3. Others (specify)________________	
204	How many rooms are used by you and your HH for sleeping only	Number of rooms_____________________	
205	Do you have kitchen?	1. Yes ☐<br>2. No ☐	
206	Do you have separate rooms for cattle and or storage use?	1. Yes ☐<br>2. No ☐	
207	What is the wall of your house made of?	1. Wooden structure ☐<br>2. Mud ☐<br>3. Cement ☐<br>4. Other (specify)__________________	
208	Do you and your HH have?		
	a. Functioning TV	1. Yes ☐ 2. No ☐	
	b. Functioning radio	1. Yes ☐ 2. No ☐	
	c. Bed and mattress	1. Yes ☐ 2. No ☐	
	2. Chair and table	1. Yes ☐ 2. No ☐	
	3. Car	1. Yes ☐ 2. No ☐	
	4. Motor bike (okada)	1. Yes ☐ 2. No ☐	

C. Health and Health Related Questions

No.	Questions	Response Code *(check the appropriate option or state nos.)*	Skip
300	How do you rate the health status of your family?	1. Very Poor ☐<br>2. Poor ☐<br>3. Medium ☐<br>4. Good ☐<br>5. Very Good ☐	
301	Do you or another member of the HH have chronic illness and/or disability?	1. Yes ☐<br>2. No ☐	
302	Have any member of the family encountered any illness during the past 3 months?	1. Yes ☐<br>2. No ☐	If "No" skip to 304
303	How many of the members were ill?	The number of the ill members__________	
304	Did you seek medical treatment for the recent episode?	1. Yes ☐<br>2. No ☐	If "No" skip to 306

No.	Questions	Response Code *(check the appropriate option or state nos.)*	Skip
305	Did you get treatment?	1. Yes ☐<br>2. No ☐	If "No" skip to 308
306	Where did you get treatment?	1. Self-treatment ☐<br>2. Local Drug Vendor ☐<br>3. Private Health Facility ☐<br>4. Public Health Center ☐<br>5. Public Hospital ☐<br>6. Traditional Healer ☐<br>7. Other (specify)_________________	
307	Why did you go there?	1. The HF was physically accessible ☐<br>2. The HF was not expensive ☐<br>3. The HF was not too crowded ☐<br>4. The health service was courteous ☐<br>5. The health service was effective ☐<br>6. Other (specify)_________________	
308	Why did you not get treatment?	1. Consider the illness is self-limiting<br>2. Not enough money<br>3. Didn't know anywhere to go<br>4. Didn't have time to go to the HF<br>5. Too far to go to HF<br>6. Other (specify)___________________<br>7. Unknown (99)	

No.	Questions	Response Code *(check the appropriate option or state nos.)*	Skip
309	What was the total health care cost of the HH for the treatment during the last 3 months?	Amount in Le______________________	
310	Who covered the health care cost?	1. Self ☐<br>2. Family ☐<br>3. Government/Free ☐<br>4. Community ☐<br>5. Others (specify)____________________	
311	How satisfied were you with the health care service and cost?	1. Very dissatisfied ☐<br>2. Dissatisfied ☐<br>3. Neutral ☐<br>4. Satisfied ☐<br>5. Very satisfied ☐	
312	How did you perceive quality of the health care service in this area?	1. Very low ☐<br>2. Low ☐<br>3. Neutral ☐<br>4. High ☐<br>5. Very high ☐	
313	How did you see finding money to pay for the health care?	1. Very difficult ☐<br>2. Difficult ☐<br>3. Not difficult ☐	If "No" skip to 315

No.	Questions	Response Code *(check the appropriate option or state nos.)*	Skip
314	If paying for medical expenses was difficult, how did you get it?	1. Drew from savings ☐<br>2. Borrowing from someone ☐<br>3. Assisted by relatives ☐<br>4. Did extra work ☐<br>5. Sell capital assets such as cows ☐<br>6. Cut back on other things, food, drinks, clothes, etc ☐<br>7. Others (specify)_______________	
315	Did you borrow any money from relatives or other people to cover medical costs within the last year?	1. Yes ☐<br>2. No ☐	If "No" skip to 317
316	How much did you borrow?	Amount in Le_______________________	
317	What is the nearest conventional health institution to your home?	1. Health center ☐<br>2. Clinic (private) ☐<br>3. Hospital (public) ☐	
318	How long does it take to reach the nearby HF from your home?	Time in minutes__________________________	
319	What form of transportation did you use to reach the location where you obtained treatment?	1. Personal Vehicle ☐<br>2. Public Transport ☐<br>3. Ambulance ☐<br>4. Motor Bike (Okada) ☐<br>5. Bicycle ☐<br>6. Others (specify)_____________________	

PLEASE EXPLAIN THE SOCIAL HEALTH INSURANCE SCHEME SCENARIO TO THE RESPONDENT BEFORE YOU ADMINISTER QUESTIONS IN SECTIONS C & D.

D. Willingness to Join the SLeSHI Scheme

No.	Questions	Response Code *(check the appropriate option or state nos.)*	Skip
400	Based on the scenario above, will you be willing to join SLeSHI when established?	1. Yes ☐<br>2. No ☐	If "No" skip to 402
401	If "Yes", why will you join?	1. It provides free access to medical care ☐<br>2. To help others ☐<br>3. For security and peace of mind in times of ill-health ☐<br>4. Facing health problems frequently ☐<br>5. Others (specify)________________	
402	Why will you not join the Scheme?	1. I do not have enough money to pay ☐<br>2. Do not need health insurance ☐<br>3. OOP charge is better ☐<br>4. Lack of trust in government programmes ☐<br>5. Lack of functional HF in our area ☐<br>6. No qualified medical personnel in the HF ☐<br>7. Contributing money for sickness in advance is a taboo ☐<br>8. Scope of illness covered by the Scheme is limited ☐<br>9. Others (specify)________________	Stop the interview here

E. Willingness to Pay for the SLeSHI Scheme

No.	Questions	Response Code *(check the appropriate option or state nos.)*	Skip
500	Now that you have agreed to join the SLeSHI Scheme, how frequently would you prefer to pay?	1. Monthly ☐<br>2. Quarterly ☐<br>3. Half-yearly ☐<br>4. Yearly ☐<br>5. Others (specify)________________	
501	Assuming you were to pay Le 20,000 per month per head as premium for the SLeSHI Scheme, will you be willing to pay?	1. Yes ☐<br>2. No ☐<br>3. Don't know ☐	If either "No" or "Don't know" skip to 503
502	If the premium is set at Le 30,000 per month per head for the SLeSHI Scheme, would you be willing to pay?	1. Yes ☐<br>2. No ☐<br>3. Don't know ☐	If either "No" or "Don't know" skip to 504
503	If the premium is set at Le 10,000 per month per head for the SLeSHI Scheme, would you be willing to pay?	1. Yes ☐<br>2. No ☐<br>3. Don't know ☐	
504	What is the Maximum Amount will you be willing to pay per head based on your choice mode of payment?	Amount in Le________________________	
505	If the answer is Le 0.00 in 401, then tell us why is you and your household not willing to pay for the Scheme?	1. I doubt whether it will be managed properly ☐<br>2. It should be the responsibility of the government ☐<br>3. Because of lack of money ☐<br>4. The wealthy people should pay for the programme ☐<br>5. Other (specify)________________	
